# Closed-Loop Tracking and Regulation of Emotional Valence State From Facial Electromyogram Measurements

**DOI:** 10.3389/fncom.2022.747735

**Published:** 2022-03-25

**Authors:** Luciano R. F. Branco, Arian Ehteshami, Hamid Fekri Azgomi, Rose T. Faghih

**Affiliations:** ^1^Department of Electrical and Computer Engineering, University of Houston, Houston, TX, United States; ^2^Department of Neurological Surgery, University of California, San Francisco, San Francisco, CA, United States; ^3^Department of Biomedical Engineering, New York University, New York, NY, United States

**Keywords:** closed-loop, control, brain, emotion, valence, electromyogram (EMG), wearable, state-space

## Abstract

Affective studies provide essential insights to address emotion recognition and tracking. In traditional open-loop structures, a lack of knowledge about the internal emotional state makes the system incapable of adjusting stimuli parameters and automatically responding to changes in the brain. To address this issue, we propose to use facial electromyogram measurements as biomarkers to infer the internal hidden brain state as feedback to close the loop. In this research, we develop a systematic way to track and control emotional valence, which codes emotions as being pleasant or obstructive. Hence, we conduct a simulation study by modeling and tracking the subject's emotional valence dynamics using state-space approaches. We employ Bayesian filtering to estimate the person-specific model parameters along with the hidden valence state, using continuous and binary features extracted from experimental electromyogram measurements. Moreover, we utilize a mixed-filter estimator to infer the secluded brain state in a real-time simulation environment. We close the loop with a fuzzy logic controller in two categories of regulation: inhibition and excitation. By designing a control action, we aim to automatically reflect any required adjustments within the simulation and reach the desired emotional state levels. Final results demonstrate that, by making use of physiological data, the proposed controller could effectively regulate the estimated valence state. Ultimately, we envision future outcomes of this research to support alternative forms of self-therapy by using wearable machine interface architectures capable of mitigating periods of pervasive emotions and maintaining daily well-being and welfare.

## 1. Introduction

Emotions directly influence the way we think and interact with others in different situations, especially when it interferes with rationality in our decision-making or perception (Dolan, 2002). Thus, having a solid grasp of the dynamics of emotions is critical to provide any therapeutic solutions to maintain welfare (Couette et al., 2020). Moreover, deciphering emotions has been an ongoing task among researchers, dictating joint efforts from behavioral, physiological, and computational angles (Scherer, 2005). According to the James A. Russell's circumplex model of affect, emotion can be divided into two perpendicular axes, viz. valence—reflecting the spectrum of negative to positive emotions—and arousal, accounting for the intensity characteristics (Russell, 1980). In this study, we focus on improving comprehension of emotional valence regulation by proposing an architecture to track and regulate the internal hidden valence state using physiological signals collected via wearable devices. The use of wearable devices to gain insight to the internal brain state provides a good alternative to study the brain dynamics, as usually the procedures either rely on invasive techniques, e.g., extracting bloodstream samples, performing surgery, or require large and expensive equipment for imaging purposes (Villanueva-Meyer et al., 2017; Wickramasuriya et al., 2019a,b).

Affective computing is defined by an interdisciplinary field of research that incorporates both sentiment analysis and emotion recognition (Poria et al., 2017). Scholars have posited the importance of affective computing to endow machines with the means to recognize, interpret or convey emotions and sentiments (Poria et al., 2017; Burzagli and Naldini, 2020). These capabilities allow the development and enhancement of personal care systems that interact better with humans, potentially improving a personal health and daily well-being (Burzagli and Naldini, 2020). Previous attempts in the development of affective computing have focused on emotion feature extraction and classification through human-robot interactions (Azuar et al., 2019; Rudovic et al., 2019; Yu and Tapus, 2019; Filippini et al., 2020; Rosula Reyes et al., 2020; Val-Calvo et al., 2020), facial expressions (Chronaki et al., 2015; rong Mao et al., 2015; Yang et al., 2018a; Zeng et al., 2018), and vocal responses (Wang et al., 2015; Fayek et al., 2017; Noroozi et al., 2017; Anuja and Sanjeev, 2020). The objective of this research is to take this one step further and introduce a tracking and closed-loop control framework to regulate specific emotions.

Within a closed-loop approach, biomarkers are collected in real-time as feedback, which grants the possibility of automatically adjusting brain stimulation levels according to the current emotional state (Wickramasuriya et al., 2019a,b; Thenaisie et al., 2021). Previous studies have shown that this strategy can increase treatment efficacy and decrease the extent of stimulation side-effects, compared to just employing an open-loop stimulation (Price et al., 2020). The benefits of closed-loop neurostimulation have been well reported in addressing conventional-therapy-resistant patients with Parkinson's disease (Little et al., 2016; Weiss and Massano, 2018). However, fewer studies have explored closed-loop therapies for non-motor neuropathologies such as post-traumatic stress disorder or depression (Tegeler et al., 2017; Mertens et al., 2018), even though there is already relevant evidence of improvements with open-loop therapies (Conway et al., 2018; Starnes et al., 2019; Freire et al., 2020). Conversely with the conventional open-loop approach, brain stimulation is manually tuned during in-clinic visits, delivering pre-determined quantities and incurring over or under stimulation of the brain (Wickramasuriya et al., 2019a,b; Price et al., 2020).

To properly regulate the emotional brain state in a closed-loop manner, a suitable biomarker that relates to the internal emotional valence needs to be identified. Prior research has based emotion classification on facial or voice expressions, which not only requires heavy data acquisition, but also runs into ambiguity issues (Tan et al., 2012). Facial and vocal expressions can vary significantly between person to person, making it difficult to draw any accurate inference about the person's emotional state. Moreover, facial and vocal expressions (e.g., smiling) can be seen as externalized emotions and can be altered at will, confounding the accuracy of such classification approaches, and thus hindering any tracking and control efforts as the true emotional state would not be clear (Cai et al., 2018). In response, our proposed strategy aims to remove this ambiguity by using a more reliable metric: physiological signals (Cacioppo et al., 2000). Physiological signals or biomarkers are involuntary responses initiated by the human's central and autonomic nervous systems, whereas facial and vocal lineaments can voluntarily be hidden to reject certain emotional displays (Cannon, 1927; Cacioppo et al., 2000; Lin et al., 2018; Amin and Faghih, 2020; Wilson et al., 2020). Although overall facial expression can be made to mask certain emotions, several studies have linked electromyogram activity of specific facial muscles to states of affection in varying valence levels, such as happiness, stress and anger (Nakasone et al., 2005; Kulic and Croft, 2007; Gruebler and Suzuki, 2010; Tan et al., 2012; Amin et al., 2016; Cai et al., 2018). Cacioppo et al. described that the somatic effectors of the face are tied to changes in connective tissue rather than skeletal complexes (Cacioppo et al., 1986). Researchers in Cacioppo et al. (1986) posited facial electromyogram could provide insight into valence state recognition even when there is no apparent change in facial expressions. Moreso, the work of Ekman et al. (1980) and Brown and Schwartz (Brown and Schwartz, 1980) are two of the few who showed that using facial electromyogram measurements of the zygomaticus muscle (zEMG) gave the most distinct indicator of valence compared to other facial muscles involved in the act of smiling. Multiple studies have suggested the relation between emotional states and facial electromyogram activity (Van Boxtel, 2010; Tan et al., 2011; Koelstra et al., 2012; Künecke et al., 2014; Kordsachia et al., 2018; Kayser et al., 2021; Shiva et al., 2021). Golland et al. (2018) also showcased a consistent relationship between the emotional media viewed and the changes seen in the components of the facial electromyogram signal. We focus on zEMG to build our model and track the hidden valence state. Then, we design a control strategy to automatically regulate the internal emotional valence state in real-time.

It should be noted that electromyogram is not the only physiological metric that has shown promise for valence recognition. Emotional valence can also be represented by many different physiological signals or a combination of them (Egger et al., 2019), such as using electroencephalography (Bozhkov et al., 2017; Wu et al., 2017; Soroush et al., 2019; Feradov et al., 2020), respiration (Zhang et al., 2017; Wickramasuriya et al., 2019a,b), electrocardiography (ECG) (Das et al., 2016; Goshvarpour et al., 2017; Harper and Southern, 2020), blood volume pulse (Das et al., 2016) or heart rate variability (Ravindran et al., 2019). Egger et al. investigated the accuracy of different physiological signals in classifying emotive states such as stress periods, calmness, despair, discontent, erotica, interest, boredom, or elation (Egger et al., 2019). Naji and collaborators displayed the disparity between multimodal and individual signal measurements regarding emotion classification via ECG and forehead biosignals (Naji et al., 2014).

Previous studies have also investigated different ways of estimating and tracking internal brain states (Sakkalis, 2011). Brain dynamics during resting states have been studied with measurements from functional magnetic resonance imaging (fMRI), using linear and non-linear models, and more recently, employing a tensor based approach (Honey et al., 2009; Abdelnour et al., 2014; Al-Sharoa et al., 2018). The transition of brain states has been examined with machine learning methods and eigenvalue decomposition, by using data from fMRI, electroencephalogram (EEG) or magnetoencephalography (Pfurtscheller et al., 1998; Guimaraes et al., 2007; LaConte et al., 2007; Maheshwari et al., 2020). Moreover, EEG measurements were also employed with machine learning techniques to estimate stress levels (Al-Shargie et al., 2015), and affection (Nie et al., 2011). The method introduced by Yadav et al. uses a state-space formulation to track and classify emotional valence based on two simultaneous assessments of brain activity (Yadav et al., 2019). In the present work, we use a similar approach to estimate and track the hidden valence state, with the help of Bayesian filtering as a powerful statistical tool to improve state estimation under measurement uncertainties (Prerau et al., 2009; Ahmadi et al., 2019; Wickramasuriya and Faghih, 2020). Another contribution of the present work is the use of real measurements from wearable devices to develop a virtual subject environment as a simulation framework for concealed emotional levels. This is the first step to empower the implementation and testing of closed-loop controllers that could track and regulate the internal valence state. In a similar fashion to other control studies, providing a reliable closed-loop simulation framework can pave the way for safe experimentation of brain-related control algorithms here and in future studies (Santaniello et al., 2010; Dunn and Lowery, 2013; Yang et al., 2018b; Wei et al., 2020; Ionescu et al., 2021).

To investigate the validity of regulating emotions through a closed-loop control architecture, we design a simulation system using experimental data. Specifically, in this *in silico* study, we employ features extracted from zEMG data and design a fuzzy logic controller to regulate the emotional valence state in a closed-loop manner. We propose to implement fuzzy logic as this knowledge-based controller works with a set of predetermined fuzzy rules and weights responsible to gauge the degree in which the input variables are classified into output membership functions (Klir and Yuan, 1995; Qi et al., 2019). This process is particularly useful for controlling complex biological systems, as it provides a simple yet effective way of interacting with the uncertainties and impreciseness of these challenging systems (Lilly, 2011). In the literature, previous research have explored the use of a fuzzy logic controller in a simulation environment to control cognitive stress or regulate energy levels of patients with clinical hypercortisolism (Azgomi and Faghih, 2019; Azgomi et al., 2019). A fuzzy controller was also combined with a classical Proportional Integral Derivative (PID) controller to aid the movement of a knee prosthesis leg (Wiem et al., 2018), and to regulate movement of the elbow joint of an exoskeleton during post-stroke rehabilitation (Tageldeen et al., 2016). Scholars have shown fuzzy logic controllers to outperform PID controllers in the regulation of mean arterial pressure (Sharma et al., 2020), and to improve the anesthetic levels of patients undergoing general anesthesia (Mendez et al., 2018). In light of what is presented, in this *in silico* study, we develop a virtual subject environment to evaluate the efficiency of our proposed architecture.

The remainder of this research is organized as follows. In Section 2 we describe the methods used in this research. Specifically, in Section 2.1 we describe the virtual subject environment and the steps taken toward its development (i.e., the models used, the features extracted, the valence state estimation and the modeling of the environmental stimuli). Next, in Section 2.2 we explain the controller design and the steps taken during implementation. Then, we present our results in Section 3, followed by a discussion of those in Section 4.

## 2. Methods

### 2.1. Virtual Subject Environment

**Figure 1:**
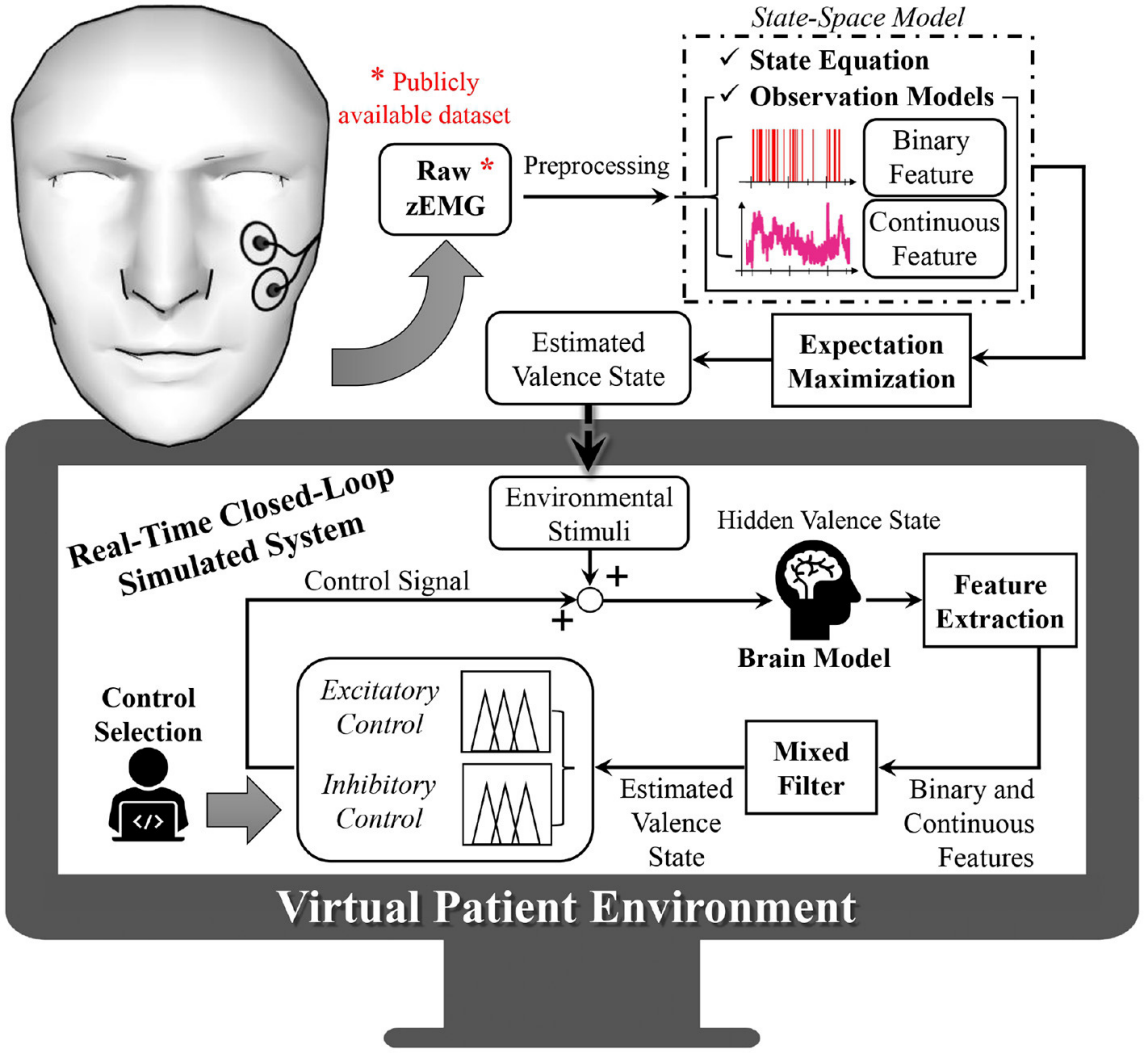
Overview of proposed closed-loop solution. Within data from a publicly available dataset, the subject is wearing wearable electromyogram sensors that collect facial muscle activity. From the electromyogram measurements, binary and continuous features are extracted and used to infer the hidden emotional valence state of the subject, which cannot be measured directly. This is performed using state-space modeling and via an expectation maximization algorithm. The estimated valence state is then used to model an environmental stimuli, recreating the subject's surrounding input inside the virtual subject environment. Within this virtual environment, different emotional conditions are recreated into the brain model. By extracting binary and continuous features and using a mixed filter, the subject's hidden emotional valence state is estimated and further regulated as desired (excitation or inhibition modes) by means of a fuzzy logic controller.

An overview of the proposed system is presented in Figure 1. As depicted in Figure 1, to construct the virtual subject environment we first take the zEMG measurements and preprocess the collected data for our further analysis. From the zEMG data we extract binary and continuous features that will be used both to build the state-space model and to estimate hidden emotional levels. This is possible after the establishment of the continuous and binary observation models associated with the state-space representation of emotional valence. Since the emotional valence progression of the subject is not measurable directly, we use the two simultaneous features and an expectation maximization (EM) algorithm, to model and drive the environmental stimuli within the virtual subject environment. The environmental stimuli are used to recreate, in real-time, different subject-specific emotional valence state-related responses into the simulated brain model. Similarly to the non-real-time case, output from the brain model will then have binary and continuous features extracted before reaching the mixed-filter. The mixed-filter estimates the hidden valence state to supply it to the control method selected of either excitatory or inhibitory control. With these two classes of closed-loop regulation we can analyze the performance of the proposed approach. Finally, the control algorithm determines the control effort necessary and provides it to the brain model, closing the loop. All the simulations of this research were performed using SIMULINK from MATLAB (The Math Works, Inc., Natick, MA) version 2020b.

#### 2.1.1. Dataset

In this research, we develop human brain models using the publicly available Database for Emotion Analysis using Physiological Signals (DEAP) (Koelstra et al., 2012), in which the authors investigated the connection between physiological signals and an associated emotional tag, based on a valence scale. In the DEAP dataset, 32 subjects (16-females and 16-males, mean age 26.9) were asked to watch 1 min segments of 40 different music videos. These videos were selected so that they would capture every aspect of both arousal and valence levels. At the end of each video trial, the researchers gathered each subject's self-assessment regarding emotional valence, on a 1–9 scale. During the experiment, various physiological signals were collected, such as the facial zEMG response at 512 Hz. For our study, the self-assessed emotional valence information is taken as ground truth.

#### 2.1.2. State-Space Model

We model the valence state progression by forming stochastic state-space models.

##### 2.1.2.1. State Equation

Similar to Prerau et al. (2009), we use a first order autoregressive state-space model,


(1) $$\begin{array}{l} x_{k+1}=x_{k}+\epsilon_{k}+s_{k}+u_{k}, \end{array}$$

where *x*_*k*_ is the hidden valence state at time step *k* for *k* = 1, …, *K* and *K* is the entire experiment duration. The model also includes the process noise as a Gaussian zero-mean random variable $\epsilon_{k}\sim N\left(0,\sigma_{\epsilon }^{2}\right)$, *s*_*k*_ as a surrogate for any environmental stimuli that influenced the brain at the time of data collection, and *u*_*k*_ as the input from the controller.

##### 2.1.2.2. Observation Models

We include two observation models that capture the evolution of the zEMG signals binary and continuous features so that we can observe the valence state progression in Equation (1). By using two features simultaneously in the model, we achieve a more accurate (i.e., narrower confidence intervals) and more precise emotional state estimation (Prerau et al., 2008). The binary observations *n*_*k*_ = {0, 1}, are modeled as a Bernoulli distribution (McCullagh and Nelder, 1989; Wickramasuriya et al., 2019a,b),


(2) $$\begin{array}{l} P\left(n_{k}|x_{k}\right)=p_{k}^{n_{k}}{\left(1-p_{k}\right)}^{1-n_{k}}, \end{array}$$

(3) $$\begin{array}{l} p_{k}=\frac{e^{\gamma +x_{k}}}{1+e^{\gamma +x_{k}}}, \end{array}$$

where *p*_*k*_ is the probability of observing a spike given the current valence state amplitude via sigmoidal link function (Equation 3), which has shown to depict frequency or counting datasets well (Wickramasuriya et al., 2019a,b). The continuous observations *z*_*k*_ ∈ ℝ are modeled as,


(4) $$\begin{array}{l} z_{k}=\alpha +\beta x_{k}+\omega_{k}, \end{array}$$

where α is a coefficient representing the baseline power of the continuous feature, β is the rate of change in the continuous feature's power, and ω_*k*_ is a normally distributed zero mean Gaussian random variable $\omega_{k}\sim N\left(0,\sigma_{\omega }^{2}\right)$. Both the continuous and binary observations are stated as functions of the valence state *x*_*k*_.

#### 2.1.3. zEMG Feature Extraction

To perform the estimation process and obtain the hidden valence state, we utilize the zEMG data and extract the binary and continuous features presented in the observation models.

##### 2.1.3.1. Data Preprocessing

We use a third order butterworth bandpass filter between 10 and 250 Hz to remove motion artifacts and other unwanted high frequency noise. Additionally, we use notch filters at 50 Hz and next four harmonics to remove any electrical line interference. Finally, the filtered zEMG signal, *y*_*k*_, is segmented into 0.5 second bins with no overlapping.

##### 2.1.3.2. Binary Feature Extraction of Filtered zEMG

As suggested by previous scholars the binary features extracted from the zEMG signal may be associated with the underlying neural spiking activity (Prerau et al., 2008; Amin and Faghih, 2020; Azgomi et al., 2021a). Thus, we estimate the neural spiking pertinent to emotional valence by extracting binary features from the zEMG data. Firstly, the bins of the filtered zEMG signal *y*_*k*_ are rectified by taking their absolute values and then smoothed with a Gaussian kernel. Similarly to Azgomi et al. (2019) and Yadav et al. (2019), the binary features *n*_*k*_ are obtained with the Bernoulli distribution,


(5) $$\begin{array}{l} P\left(n_{k}|y_{k}\right)=q_{k}^{n_{k}}{\left(1-q_{k}\right)}^{1-n_{k}}, \end{array}$$

(6) $$\begin{array}{l} q_{k}=a\;y_{k}, \end{array}$$

where *a* is a scaling coefficient, chosen heuristically to be 0.5, and *q*_*k*_ is a zEMG amplitude dependent probability function of observing a spike in bin *k*, given *y*_*k*_.

#### 2.1.4. Continuous Feature Extraction of Filtered EMG

Using the filtered zEMG signal *y*_*k*_, we also extract the continuous features employing the Welch power spectral density (PSD) of each 0.5 s bin, with a 75% window overlap. Afterwards, for each bin, we compute the bandpower of the PSD result from 10 to 250 Hz, before taking the logarithm. Finally, we normalize the entire signal on a 0–1 scale, to provide insight of the relative band power of the zygomaticus major muscle activity, across all 40 1-min trials.

#### 2.1.5. Hidden Valence State Estimation

To estimate the emotional valence fluctuations within the experimental data, we employ the state-space representation shown in Equation (1) without the control effort and environmental stimuli, since at this time, there is no control signal and the stimuli is inherent in the data. The hidden valence state process is defined by


(7) $$\begin{array}{l} x_{k+1}=x_{k}+\epsilon_{k}. \end{array}$$

Given the complete values for both extracted binary *N*_1:*K*_ = {*n*_1_, …, *n*_*K*_} and continuous *Z*_1:*K*_ = {*z*_1_, …, *z*_*K*_} features, we use the EM algorithm to estimate the model parameters θ = [α, β, σ_ϵ_, σ_ω_] and the hidden valence state *x*_*k*_. The EM algorithm provides a way to jointly estimate the latent state and parameters of the state-space models. Composed of two steps, namely, Expectation step (E-step) and Maximization step (M-step), the EM algorithm: (1) finds the expected value of the complete data log-likelihood, and (2) maximizes the parameters corresponding to this data log-likelihood. The algorithm iterates between these two steps until convergence (Wickramasuriya et al., 2019a,b; Yadav et al., 2019). The following equations show how at iteration (*i* + 1) values are recursively predicted with estimates and parameters from iteration *i* (e.g., $x_{0}^{\left(i\right)}$, $\sigma_{\epsilon }^{2\left(i\right)}$).

##### 2.1.5.1. E-Step

###### 2.1.5.1.1. Kalman-Based Mixed-Filter (Forward-Filter).


(8) $$\begin{array}{l} x_{k|k-1}=x_{k-1|k-1} \end{array}$$

(9) $$\begin{array}{l} \sigma_{k|k-1}^{2}=\sigma_{k-1|k-1}^{2}+\sigma_{\epsilon }^{2\left(i\right)} \end{array}$$

(10) $$\begin{array}{l} C_{k}={\left(\beta^{\left(i\right)2}\sigma_{k|k-1}^{2}+\sigma_{\omega }^{2\left(i\right)}\right)}^{-1}\sigma_{k|k-1}^{2} \end{array}$$

(11) $$\begin{array}{l} \begin{array}{c} {\hat{x}}_{k}=x_{k|k}=x_{k|k-1}+C_{k}[\beta^{\left(i\right)}(z_{k}-\alpha^{\left(i\right)}\\ \;-\beta^{\left(i\right)}x_{k|k-1})+\sigma_{\omega }^{2\left(i\right)}(n_{k}-p_{k|k})] \end{array} \end{array}$$

(12) $$\begin{array}{l} \begin{array}{c} {\hat{\sigma }}_{k}^{2}=\sigma_{k|k}^{2}=[{\left(\sigma_{k|k-1}^{2}\right)}^{-1}+p_{k|k}\left(1-p_{k|k}\right)\\ \;+{\left(\sigma_{\omega }^{2\left(i\right)}\right)}^{-1}\beta^{\left(i\right)2}{]}^{-1} \end{array} \end{array}$$

where *k* = 1, …, *K*; ${\hat{x}}_{k}$ is the estimated valence state; and ${\hat{\sigma }}_{k}^{2}$ constitute the corresponding standard deviation.

###### 2.1.5.1.2. Fixed-Interval Smoothing Algorithm (Backward-Filter).


(13) $$\begin{array}{l} A_{k}=\sigma_{k|k}^{2}(\sigma_{k+1|k}^{2}{)}^{-1} \end{array}$$

(14) $$\begin{array}{l} x_{k|K}=x_{k|k}+A_{k}\left(x_{k+1|K}-x_{k+1|k}\right) \end{array}$$

(15) $$\begin{array}{l} \sigma_{k|K}^{2}=\sigma_{k|k}^{2}+A_{k}^{2}\left(\sigma_{k+1|k}^{2}-\sigma_{k+1|K}^{2}\right) \end{array}$$

###### 2.1.5.1.3. State-Space Covariance Algorithm.


(16) $$\begin{array}{l} \sigma_{k,u|k}=A_{k}\sigma_{k+1,u|k} \end{array}$$

(17) $$\begin{array}{l} W_{k|K}=\sigma_{k|K}^{2}+x_{k|K}^{2} \end{array}$$

(18) $$\begin{array}{l} W_{k-1,k|K}=\sigma_{k-1|K}+x_{k-1|K}x_{k|K} \end{array}$$

for 1 ≤ *k* ≤ *u* ≤ *K*.

##### 2.1.5.2. M-Step


(19) $$\begin{array}{l} x_{0}^{\left(i+1\right)}=x_{1|k} \end{array}$$

(20) $$\begin{array}{l} \begin{array}{c} \sigma_{\omega }^{2\left(i+1\right)}=K^{-1}\overset{K}{\underset{k=1}{\sum }}z_{k}^{2}+K\alpha^{2\left(i+1\right)}\\ \;+\beta^{2\left(i+1\right)}\overset{K}{\underset{k=1}{\sum }}W_{k|K}-2\alpha^{\left(i+1\right)}\overset{K}{\underset{k=1}{\sum }}z_{k}\\ \;-2\beta^{\left(i+1\right)}\overset{K}{\underset{k=1}{\sum }}x_{k|K}z_{k}\\ \;+2\alpha^{\left(i+1\right)}\beta^{\left(i+1\right)}\overset{K}{\underset{k=1}{\sum }}x_{k|K} \end{array} \end{array}$$

(21) $$\begin{array}{l} \begin{array}{c} \left[\begin{array}{c} \alpha^{\left(i+1\right)}\\ \beta^{\left(i+1\right)} \end{array}\right]={\left[\begin{array}{cc} K & \sum_{k=1}^{K}x_{k|K}\\ \sum_{k=1}^{K}x_{k|K} & \sum_{k=1}^{K}W_{k|K} \end{array}\right]}^{-1}\\ \;\times \left[\begin{array}{c} \sum_{k=1}^{K}z_{k}\\ \sum_{k=1}^{K}x_{k|K}z_{k} \end{array}\right] \end{array} \end{array}$$

(22) $$\begin{array}{l} \sigma_{\epsilon }^{2\left(i+1\right)}=K^{-1}\overset{K}{\underset{k=1}{\sum }}\left[W_{k|K}-2W_{k-1,k|K}+W_{k-1|K}\right] \end{array}$$

#### 2.1.6. Environmental Stimuli Model

We model the environmental stimuli referred to in Eqouation (1) as a way to capture and recreate the subject's response to high or low valence trials. This allows for the simulation of subject-specific HV and LV conditions. The environmental stimuli are calculated by finding the difference between adjacent elements of the estimated valence state ${\hat{x}}_{k}$, as in


(23) $$\begin{array}{l} s_{k}={\hat{x}}_{k+1}-{\hat{x}}_{k} \end{array}$$

for *k* = 1, …, *K*−1. Then, we assume a sinusoidal harmonic formulation to model the environmental stimuli in either HV or LV trials,


(24) $$\begin{array}{l} s_{k}=\overset{100}{\underset{j=1}{\sum }}\rho_{j}sin\left(\zeta_{j}k+\phi_{j}\right) \end{array}$$

**Figure 2:**
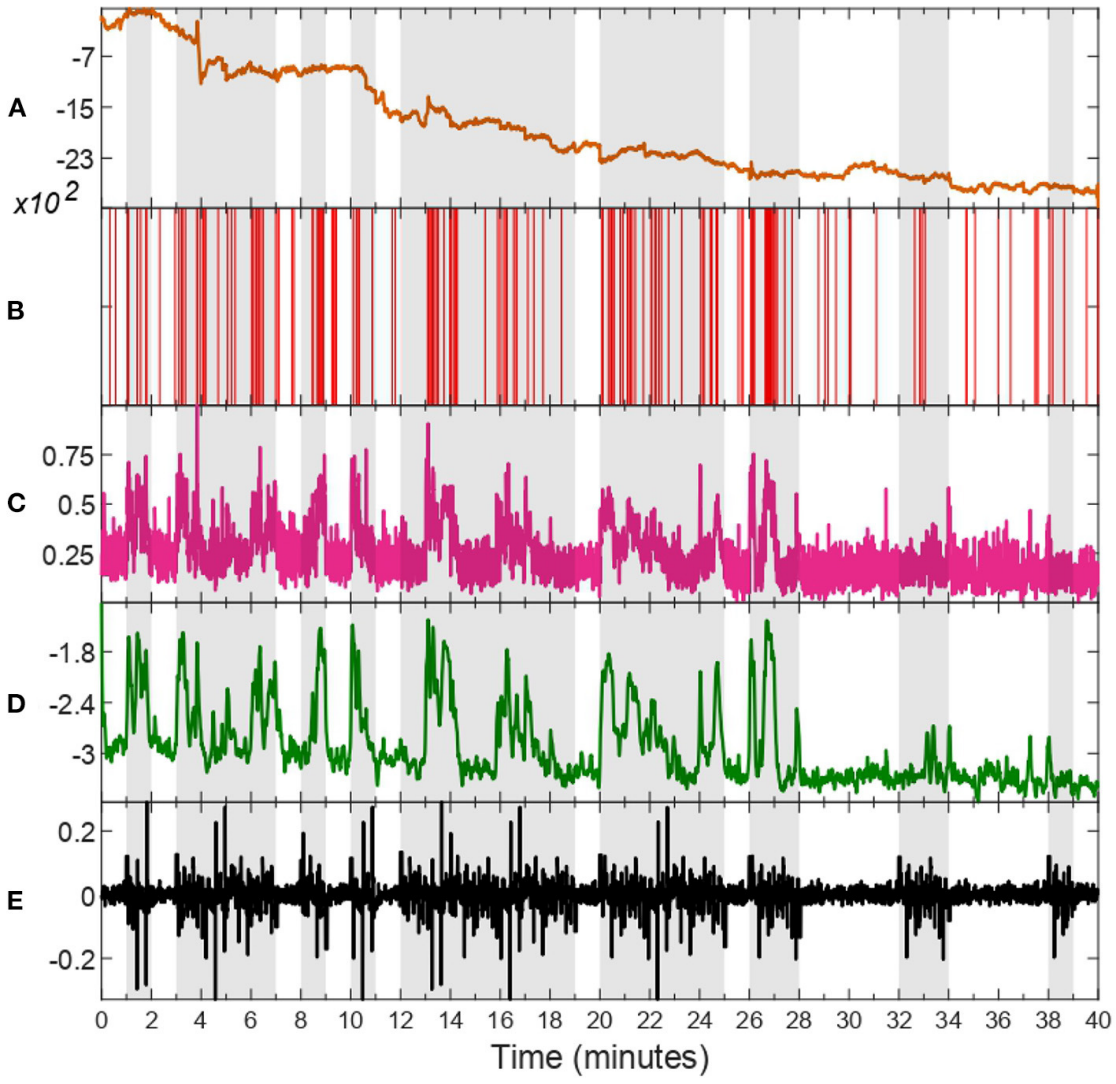
zEMG data, corresponding features, estimated valence state, and environmental stimuli of subject 18. Trials characterized as high valence (HV) are shaded in gray, whilst unshaded ones as representative of low valence (LV). The raw zEMG collected is presented in **(A)** in orange, while **(B,C)** show the extracted features, binary (red) and continuous (pink), respectively. **(D)** illustrates the hidden valence state (green) attained with the EM algorithm by employing both features shown in **(B,C)**. The last **(E)** shows the environmental stimuli (black) obtained from the valence state progression in **(D)**.

**Figure 3:**
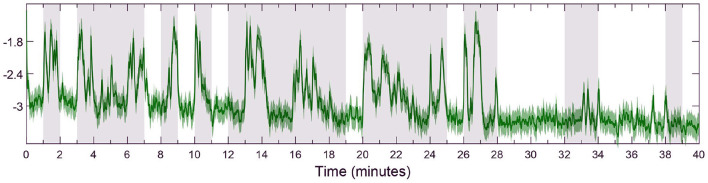
Detail of estimated emotional valence state for subject 18 with 95% confidence intervals. The white background depicts LV periods while the gray-shaded areas show HV results. The solid green line shows the estimated valence state while the green region around it is a 95% confidence interval.

Through inspection across all subjects, we notice that HV trials tend to have a higher mean and standard deviation compared to LV ones. Thus, to avoid fitting outliers to the harmonic model depicted in Equation (24), we select the six trials with highest mean and standard deviation of estimated valence levels for fitting *s*_*k*_ to HV, and the six trials with the lowest mean and standard deviation to model LV periods. Additionally, we consider a transition period between each different valence state, as approximated by a linear relationship of 0.5 s in duration. This is done separately for each subject to ensure personalized models. Data from an exemplary subject is depicted in Figure 2, in which every step of the process is illustrated separately, i.e., raw zEMG to extracted features and valence state and finally obtaining a corresponding environmental stimuli. In addition, in Figure 3, the estimated emotional valence state for the same exemplary subject is presented with 95% confidence intervals. Of the 23 subjects available in the dataset, we excluded five participants due to a lack of emotional response found when comparing between LV and HV periods, that is, both emotional periods have shown equivalent outcomes regarding both features and estimated valence state.

### 2.2. Closed-Loop Control Design

**Figure 4:**
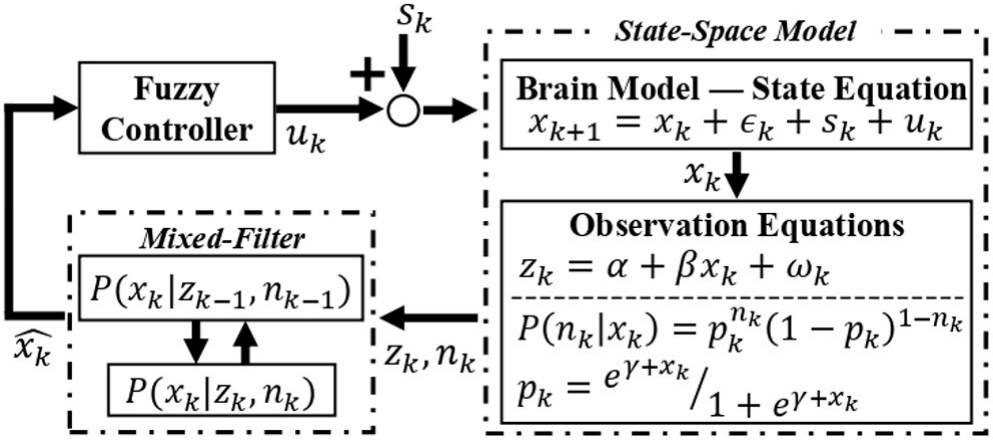
Overview of the closed-loop solution. The environmental stimuli *s*_*k*_ is added to the control signal *u*_*k*_ to form an input of the state-space brain model. The internal emotional valence state *x*_*k*_ is governed by the state equation and by employing the observation equations, binary and continuous features are extracted and taken in by the recursive mixed-filter. The filter estimates and tracks the hidden brain state ${\hat{x}}_{k}$, supplying this signal for the controller. Finally, the controller takes the current estimated valence state and generates a control signal *u*_*k*_ back to the brain model, thus closing the loop. This control signal is responsible for changing the valence state in the desired direction, i.e., increasing if excitatory or decreasing if inhibitory action.

With the virtual subject environment in place, we explore the regulation of emotional valence. Similar to the feature extraction process, we simulate the binary and continuous responses simultaneously from the internal brain state. In other words, we use Equations (2)–(4) to recreate within the virtual subject environment what would be inherent to the zEMG data in the real world. Then, these two features are fed to a Kalman-based mixed-filter to estimate the hidden valence state in an online fashion. The estimated state is averaged out in a 10-s window to smooth any abrupt changes before reaching the fuzzy controller, which then derives the control effort *u*_*k*_ in real-time. A diagram of the closed-loop is depicted in Figure 4. As the hidden valence state cannot be measured directly, we use the recursive, Kalman-based mixed-filter to estimate the latent valence state inherent to the brain model as detailed in Equations (8)–(12). As shown in Figure 4, this filter takes in both binary and continuous observations to compute the prior distribution using a Chapman-Kolmogrov equation, then finds the measurement likelihood via Bayes theorem, which can be summarized with, respectively,


(25) $$\begin{array}{l} p\left(x_{k}|n_{k-1},z_{k-1}\right), \end{array}$$

and


(26) $$\begin{array}{l} p\left(x_{k}|n_{k},z_{k}\right). \end{array}$$

#### 2.2.1. Fuzzy Control

**Figure 5:**
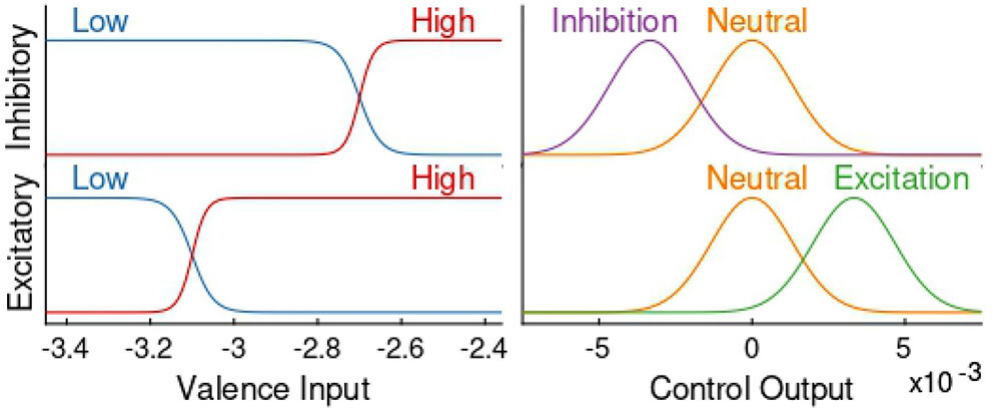
Excitatory and inhibitory fuzzy membership functions. The left side shows membership functions of the controller's input, whilst the right side display the ones for the output. The top and bottom row depict, respectively, membership functions of the inhibitory and excitatory controllers. In all four graphs the *y* axis depicts the degree of membership for every case, in which the lowest value is zero association with that function and the highest value is total association.

We use a Mamdani-type fuzzy logic controller with the fuzzy rules shown in Table 1 to regulate the subject's emotional valence to a more desired level, i.e., during inhibitory mode of control action, the goal is to achieve and remain in the same valence level characterized by the LV period—and vice-versa for the excitatory controller. As it can be observed in Figures 1, 4 and Table 1, the input signal for the controller is the estimated valence state and not a prediction error as it is more common in control studies. After analyzing the open-loop response of all subjects we designed a set of membership functions capable of directly regulating the emotional valence without subtracting it from a target reference. With this, we could employ more intuitive membership functions as depicted in Figure 5. Similarly to previous authors (Azgomi et al., 2021b), the fuzzy output can be obtained with,


(27) $$\begin{array}{l} \mu_{mamdani}\left(k\right)=\mu_{m}\left(k\right)=\underset{j}{\text{max}}\left(\text{min}\left(\mu_{valence}\left(v\right)\right)\right) \end{array}$$

where *j* designate the active rule at each time step *k* and μ_*valence*_ is the fuzzified valence input *v*. The crisp output of the fuzzy controller, i.e., the control signal *u*_*k*_, is attained using the *centroid* method as follows,


(28) $$\begin{array}{l} u_{k}=\frac{\int \mu_{m}\left(k\right).kdk}{\int \mu_{m}\left(k\right)dk}. \end{array}$$

**Table 1:** Fuzzy controller rule base.

**Input (IF):** **valence levels**	**Inhibitory** **Output (THEN):** **control action**	**Excitatory** **Output (THEN):** **control action**
Low valence	Neutral	Excitation
High valence	Inhibition	Neutral

With a fuzzy logic controller, crisp input values are transformed to degrees of membership of certain functions called membership functions in the *fuzzification* process. Then, using the pre-determined fuzzy rules the *fuzzy inference* process takes place, in which a connection between all *fuzzified* inputs is made. This results in degrees of membership of a set of output membership functions, which are then *defuzzified* to produce a final representative crisp value (Qi et al., 2019). This fuzzy logic process is convenient when dealing with complex systems, such as those biological in nature, since it allows for the emergence of complex control behaviors using relatively simple constructions (Lilly, 2011).

## 3. Results

**Figure 6:**
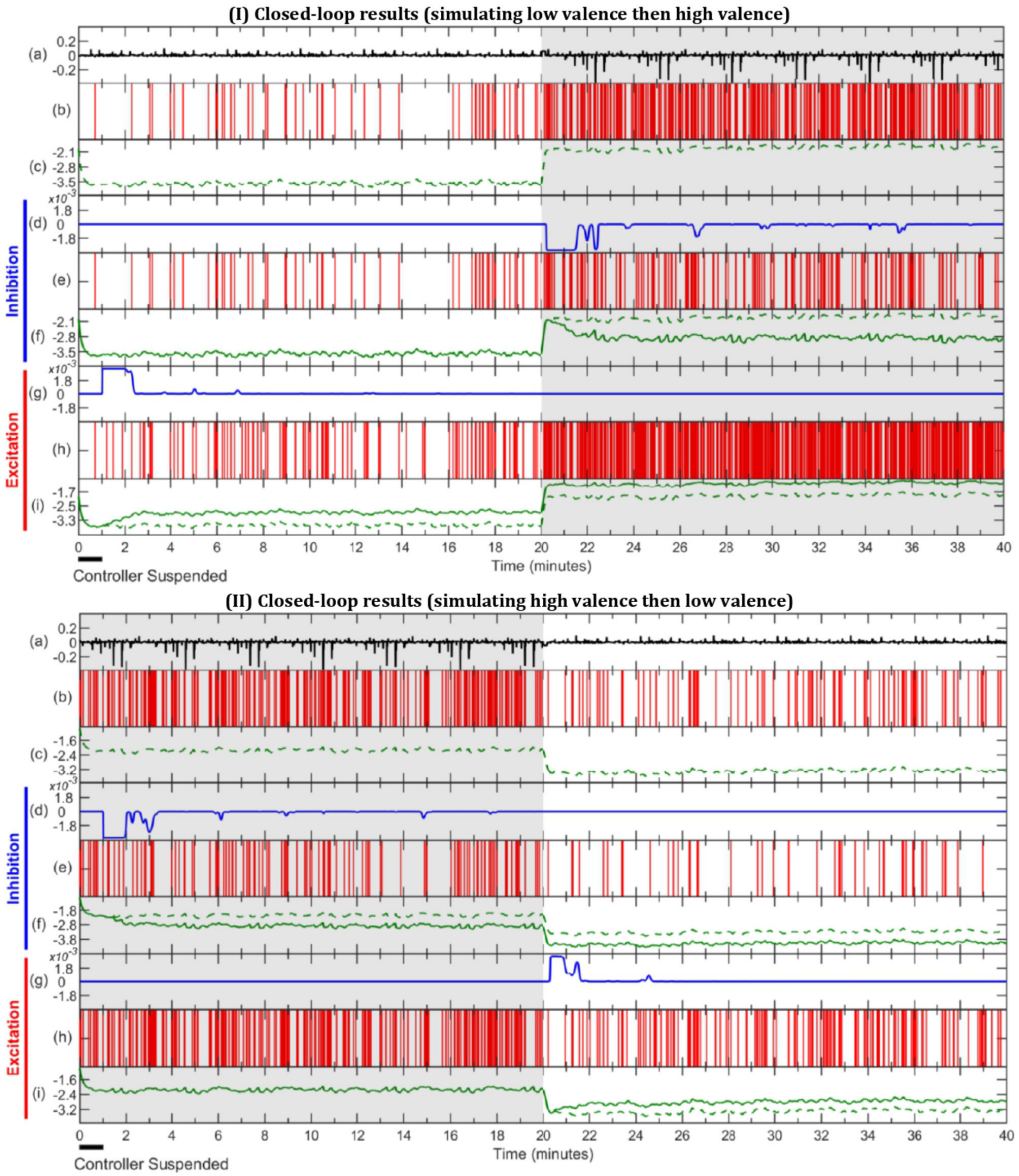
Simulation results of open-loop, inhibitory closed-loop and excitatory closed-loop scenarios for subject 7. In sub-figure I the external stimulus is comprised of half LV, then half HV, with sub-figure II being the opposite. In both **(I,II)**, LV, and HV periods are represented with unshaded and gray-shaded areas, respectively. (a) depicts environmental stimulus (black) used in all three simulation scenarios. The (b,c) show spike activity (red) and estimated valence state (green, dashed) during the open-loop, respectively. (d–f) display inhibitory closed-loop results, with (d) showing control effort (blue), (e) the corresponding binary signal (red) and (f) the comparison between open-loop (green, dashed) and closed-loop (green, solid) valence state. In a similar fashion, (g–i) exhibit the excitatory closed-loop outcome.

In this section, we present the results obtained for subject 7 in three different simulation scenaria: open-loop, inhibitory closed-loop, and excitatory closed-loop. The results associated with other subjects are also available in the Supplementary Material. We simulate with an environmental stimulus that is either half LV then half HV or vice versa. During the first minute, the controller is suspended to let the mixed-filter converge. The results are presented in Figure 6. As depicted in sub-panel (a) of I and II in Figure 6, all three scenarios for one particular subject have the same environmental stimuli in common, either starting with LV or with HV.

**Scenario 1 - Open-Loop:** Since in the open-loop scenario there is no control effort (*u*_*k*_ = 0), it can be omitted and the results are shown within the spike activity, depicted in sub-panel (b), and the corresponding estimated internal state depicted in sub-panel (c) and in dashed lines in both (f) and (i) sub-panels. It is observed, in sub-figure I of Figure 6, that the estimated valence state increases from the period of LV in the first half to HV in the second half, and so does the frequency of spikes. In contrast, sub-figure II of Figure 6 shows valence levels and number of spikes declining from the first half (HV) to the second half (LV).

**Scenario 2 - Inhibitory Closed-Loop:** The inhibitory results are observed in sub-panels (d, e, f) of both I and II in Figure 6. The control signal is zero during the LV periods of the simulations (i.e., during the first half in I and for the second half in II). It is not until the controller detects a HV period that the control effort takes a negative value (*u*_*k*_ < 0) to inhibit the emotional valence, effectively lowering the number of spikes shown in sub-panel (e) and the estimated valence state depicted in sub-panel (f), as compared to the open-loop case.

**Scenario 3 - Excitatory Closed-Loop:** The last 3 sub-panels (g, h, i) from both I and II of Figure 6 depict the results of the excitatory controller. From sub-panel (g), we can see there is no control effort in periods of HV; both in the second half of I and first half of II. Once the controller detects a low valence state, it outputs a positive control effort (*u*_*k*_ > 0), which increases the number of spikes and estimated valence level in sub-panels (h) and (i), as compared to the open-loop.

## 4. Discussion and Conclusions

In this study, we use experimental data to build a virtual subject environment, allowing us to simulate and regulate emotional valence levels using a state-space brain model and a fuzzy logic feedback controller. To the best of our knowledge, in this *in silico* feasibility study, we present the first closed-loop control framework for emotional valence state using biofeedback from facial muscles. We use two simultaneous observation models, one binary and one continuous, to relate zEMG measurements to the hidden emotional valence state. The valence state is assumed to be governed by a state-space formulation and is converted from a 1 to 9 valence scale obtained from the self-assessment of subjects from the dataset, to the high (above 5) or low valence level used in this study. These valence labels were previously used by scholars as ground truth and were also employed here to determine subject-specific simulation parameters (Yadav et al., 2019). This was done by selecting specific LV and HV trials for modeling based on a trend in the mean and standard deviation of the estimated valence state between the two categories. To capture the surrounding stimuli influencing the affective levels of the subject and incorporate them into simulation, we use the estimated emotional valence progression and a high-order harmonic formulation. This modeling and simulation of the environmental stimuli is currently necessary to evoke representative subject-specific emotional valence responses within the simulated brain model. Thus, modern control techniques can be systematically investigated *in silico*, allowing for the development of this research field without risking harm to any patients.

In the current stage of this research on closed-loop emotional valence regulation, we focus our contributions on developing the closed-loop simulated framework and opted for using a fuzzy logic controller to regulate the estimated valence state in simulated profiles. While the accuracy of the classification method is paramount for the success of our method, we employed the same methodology for classifying between low valence and high valence states which reported a 89% accuracy in previous works (Yadav et al., 2019). This value is on par with other state-of-the-art methods however, relying on physiological measurements and estimation of the brain state, instead of externalized facial or vocal expressions.

Using the proposed knowledge-based controller we successfully verify the *in silico* feasibility of the presented methods. By employing a set of simple logic rules the fuzzy system is capable of producing complex regulating behaviors (Lilly, 2011). This is extremely valuable since insight about the system can come in many ways, such as from doctors, other researchers, or the individual itself. Moreover, the fuzzy structure allows for an uncomplicated expandability feature which means other physiological signals could be simply incorporated while designing the control systems (Azgomi et al., 2021a,b). This could further enhance the approach for valence regulation.

In previous research for closed-loop regulation of human-related dynamics, scholars have developed simulators to explore controller designs for Parkinson's disease, cognitive stress, depression and other neurological and neuropsychiatric disorders, as well as for anesthetic delivery, hemodynamic stability, and mechanical ventilation (Boayue et al., 2018; Yang et al., 2018b; Azgomi et al., 2019; Parvinian et al., 2019; Fleming et al., 2020; Ionescu et al., 2021). Here, the proposed architectures set initial steps for a future wearable machine interface (WMI) implementation, as we achieved simulation of emotional valence controllers for both inhibitory and excitatory goals, demonstrating great potential in helping individuals maintain daily mental well-being (Azgomi and Faghih, 2019). While no commercial wearable solution for facial EMG measurement is available yet, the potential for this non-invasive procedure to regulate mental states encourages future efforts.

During excitatory action, we observe an increase in number of spikes and overall emotional valence state when needed and, for inhibition, our approach obtained less spikes and a lower valence level as the need arose. However, the amount of response varied with each subject due to a few reasons. One factor can be attributed to the use of a single mono-objective fuzzy controller design, in which the controller can act locally in the first half of the experiment, correctly adjusting the mental state, without considering that the environmental stimuli are going to further push the subject's valence level in the second half. This architecture also does not account for each individual peculiarities, i.e., lack or abundance of emotional engagement throughout the experiment. Further research needs to explore the optimization of fuzzy membership functions, to adapt for different persons and variations in time. Because the performance of fuzzy logic controllers are highly dependent on their parameters and structure, optimization algorithms could also improve the overall results as the parameters would not rely on pre-determined knowledge of the system (Qi et al., 2019).

In the exemplary subject depicted in Figure 6 we can observe an inhibitory action taking place in the HV periods of inhibition simulation and lowering of the number of spikes and estimated valence level as compared to the open-loop. Similarly, we can observe the excitatory controller acting in LV periods and increasing the spike frequency and valence levels, accordingly. Overall, subjects 3–5, 8, 11, and 17 (Supplementary Figures S4–S6, S9, S12, S18) showed similar results to the exemplary subject depicted in Figure 6, accomplishing reasonable regulation across all scenarios. Of the remaining 10 subjects, 7 had good performance in all inhibitory scenarios (Supplementary Figures S2, S3, S10, S11, S13, S16, S17) while 4 out of 10 had good performance in at least one excitatory scenario (Supplementary Figures S10, S14-S16). This could suggest that HV regulation is more challenging possibly due to the high variability nature of this mental state.

**Figure 7:**
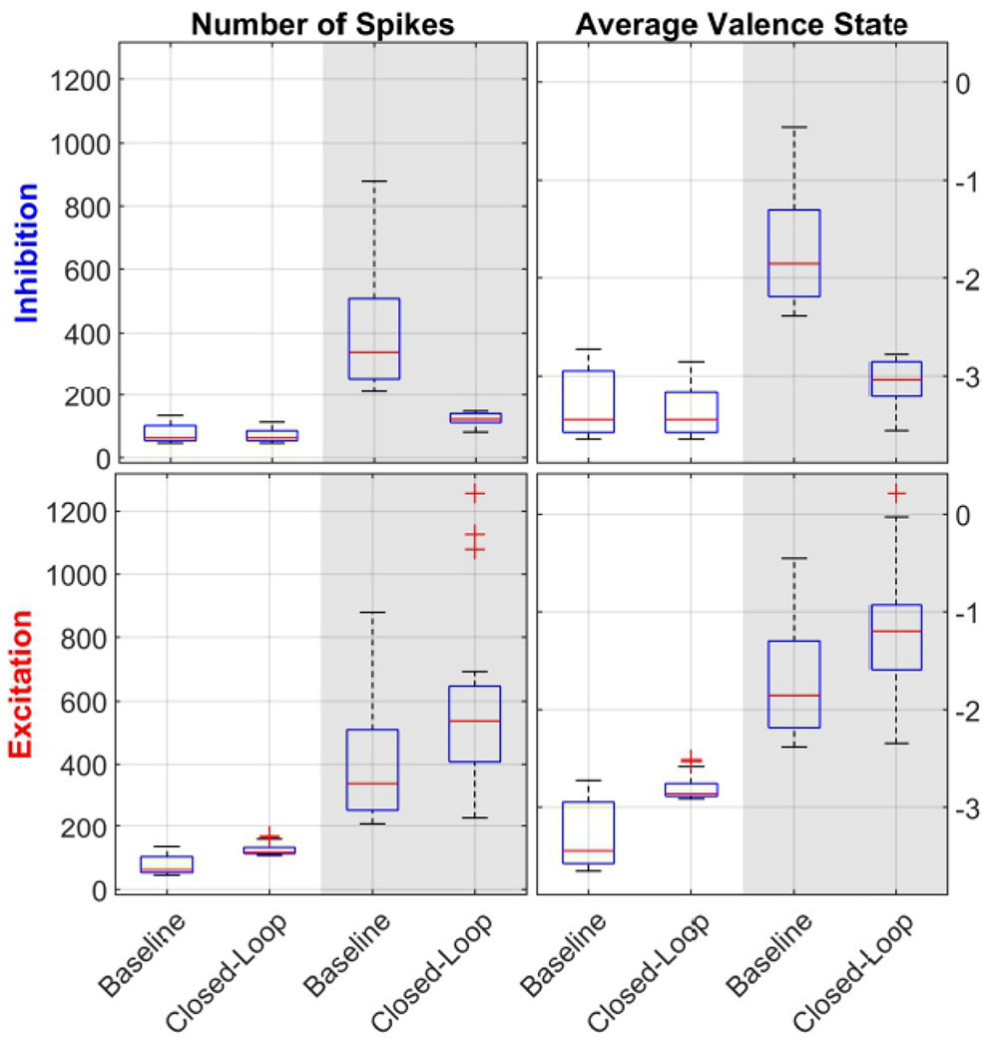
Statistical analysis with boxplot (*N* = 17) visualization of LV then HV environmental stimuli order. The left column of sub-panels shows the number of spikes in a given period, while the right column of sub-panels depicts the average valence state. The top row of sub-panels show results from the inhibitory controller and the bottom one for the excitatory one. Within each sub-plot, the white background depicts LV periods while the gray-shaded areas show HV results. Each pair of data (i.e., baseline and closed-loop) was used during the *t*-tests analysis. Comparing the open-loop baseline and closed-loop results of number of spikes and average valence levels, HV periods are statistically significant both in inhibition and excitation (all sub-panels, gray background). For LV periods, results are statistically significant only for inhibition (bottom-row, white background).

In addition to the subject exemplified in Figure 6, *t*-test analysis between the open- and closed-loop simulations with 17 out of 23 subjects was performed, as detailed in Table 2. Additionally, Figure 7 displays the distribution of data used during the *t*-test for the case of LV then HV order of environmental stimuli. The HV then LV order is also included in the Supplementary Material and presents a similar analysis. As seen both in Figure 7 and Table 2, the results show LV periods to be significantly different during excitatory action and HV trials to be significantly different throughout inhibition, regarding both the average valence level and number of spikes. This can be an indicative that the proposed controller was able to perform as desired and alter the emotional state of various subjects when required. In a similar manner, LV periods were not significantly different during inhibitory regulation if the LV was at the beginning of simulation (both in spike count, and mean valence levels). Comparing HV periods throughout excitation, the number of spikes was not significantly different when the HV period happen before LV. These results are indicative that the controller is able to detect when changes to the brain state are not required. The reason the affective state is significantly different in the second half of the experiment in cases it was not necessary (LV inhibition and HV excitation) is due to the fact that the proposed controller is not multi-objective and a regulation goal is selected beforehand | either to excite or to inhibit. Thus, after properly adjusting the brain state in the first half of the simulation, the second half will be different in comparison to the open-loop baseline and the mono-objective nature of this approach is incapable of addressing the matter. Further research is still required.

**Table 2:** Statistical analysis-*p*-values.

		**LV then HV**		**HV then LV**	
		**Number of ** **spikes**	**Average** **valence**	**Number of** **spikes**	**Average** **valence**
Inhib.	LV	0.1689	0.1947	**2 × 10^−6^**	**6 × 10^−6^**
	HV	**2 × 10^−5^**	**5 × 10^−7^**	**3 × 10^−4^**	**1 × 10^−5^**
Excit.	LV	**7 × 10^−6^**	**8 × 10^−6^**	**3 × 10^−5^**	**3 × 10^−5^**
	HV	**4 × 10^−5^**	**5 × 10^−6^**	0.0554	0.0398

*Bold values are significant p-values*.

A few subjects (4, 11, 12, 20, 21, and 23) had poor emotional valence state estimation and were discarded from the statistical analysis which also show directions for improving the proposed approach. These participants showed similar number of spikes and valence levels, during both LV and HV periods, within the open-loop scenario. Thus, when taken to a closed-loop solution, the fuzzy controller is impaired from distinguishing high and low valence levels and leads to unsatisfactory results. However, this poor valence estimation could be due to many factors such as the person not being emotionally engaged during the original data collection or distracted during the experiment (Chaouachi and Frasson, 2010). Similarly to previous scholars (Yadav et al., 2019), we investigate the performance of the emotional valence estimation with a 95% confidence intervals metric, as depicted in Figure 3. As it can be observed in Figure 3, the confidence intervals reside close to the actual recovered state and further validate the proposed state-space estimation procedure. Moreover, it is possible that these discarded subjects required additional physiological measurements (e.g., electrocardiogram, skin conductance, pupil size) to improve the estimation of the internal brain state. As mentioned above, the flexibility of the proposed state-space and fuzzy logic controller framework could easily incorporate additional physiological signals.

The present study has a few limitations. The dataset used had conflicting metadata on 9 of the 32 subjects, resulting in an impossibility of recovering the position of all 40 trials and thus, these subjects had to be discarded. Additionally, in real-world scenarios as in the dataset used, emotional valence has a spectrum of levels, but we assume only two possible states of high and low valence. This decision also reflects in the controller design in which we experiment with only two classes of closed-loop regulation, i.e., excitation and inhibition. Even with this limitation, it should be noted that both the mixed-filter and designed control provide continuous estimation and control objectives allowing for a finer regulation within this spectrum of emotions. This can be addressed in future research. Moreover, this simulation study does not incorporate the controller dynamics and real-world actuators. To implement the proposed architectures in real-world scenarios, it is paramount to consider how valence needs to be modulated, not only in terms of which actuators to use but also how frequent should interventions take place. These are challenging to address, especially when dealing with such a complex organ as the human brain, and require further investigation. In that sense, future human subject experiments shall be designed to explore the dynamics of possible actuation methods to regulate valence states. Previous scholars have observed emotional brain responses from changes in lighting or music (Schubert, 2007; Vandewalle et al., 2010; Droit-Volet et al., 2013). These would be interesting to investigate since they are also non-invasive procedures and could be incorporated in a practical system. Future research into using adaptive and predictive control strategies would also be beneficial to address some of the biological intrinsic variations of an individual. Similarly, the applicability of the proposed approach in the real world depends on the real-time estimation of mental states. At this time, we illustrate the feasibility of the approach by incorporating a simulation of brain responses on a per individual basis. Once implemented, this simulation is no longer required. However, a “training" session might be necessary to calibrate the system for each subject's peculiarities. In addition, robust state estimation or robust control design can be of tremendous importance for a real-world application. Lastly, we extracted features from LV and HV trials from EMG signal of the Zygomaticus major facial muscle, which has been depicted as a good indicator of valence (Brown and Schwartz, 1980; Ekman et al., 1980; Tan et al., 2012). As a future direction of this research, an investigation to quantify the performance in detecting fake emotional expressions via the zEMG signal would be beneficial to further enhance the proposed approach to be implemented in real life.

Using the proposed architecture, we were able to regulate one's emotional state, specifically emotional valence levels, by implementing a fuzzy controller that acted on a state-space model of the human brain. With a similar approach, a WMI could, in the future, be used to recommend a specific music track for a person feeling down, advise a change in lighting for someone in a bad mental state, or even offer a cup of green tea if the user wants to maintain a desired level of well-being (Athavale and Krishnan, 2017; Cannard et al., 2020). While we used experimental data to design a closed-loop system for regulating an internal valence state in a simulation study, a future direction of this research would be designing human subject experiments to close the loop in real-world settings. In our future work, we plan to validate the valence state estimator in real-time and close the loop accordingly. For example, we plan to incorporate safe actuators such as music or visual stimulation to close the loop. More research is needed but this suggests an important new step toward new clinical applications and the self-management of mental health.

## Data Availability Statement

The publicly available dataset used in this study can be found in http://www.eecs.qmul.ac.uk/mmv/datasets/deap/.

## Author Contributions

RF conceived and designed the study. LB, AE, HA, and RF developed the algorithms, analysis tools, and revised the manuscript. LB and AE performed research, analyzed data, and wrote the manuscript. All authors contributed to the article and approved the submitted version.

## Funding

This work was supported in part by NSF CAREER Award 1942585–MINDWATCH: Multimodal Intelligent Noninvasive brain state Decoder for Wearable AdapTive Closed-loop arcHitectures and NSF grant 1755780–CRII: CPS: Wearable-Machine Interface Architectures, and NYU start-up funds.

## Conflict of Interest

The authors declare that the research was conducted in the absence of any commercial or financial relationships that could be construed as a potential conflict of interest.

## Publisher's Note

All claims expressed in this article are solely those of the authors and do not necessarily represent those of their affiliated organizations, or those of the publisher, the editors and the reviewers. Any product that may be evaluated in this article, or claim that may be made by its manufacturer, is not guaranteed or endorsed by the publisher.
