# Supplementary material for: Closed-Loop Tracking and Regulation of Emotional Valence State From Facial Electromyogram Measurements
Source: Front Comput Neurosci. 2022 Mar 25;16:747735. doi: 10.3389/fncom.2022.747735 (PMC8990324; doi:10.3389/fncom.2022.747735)
Supplement: Supplementary file 1 [file Data_Sheet_1.PDF]

Supplementary Material

1 SUPPLEMENTARY FIGURES

Additional closed-loop simulation results from subjects (1-3; 5-10; 13-19; 22) are presented in Figs. S2-S18. The results for subjects that were discarded from statistical analysis (4, 11, 12, 20, 21, 23) are depicted in Figs. S19-S24.

Boxplot visualization of the results for high valence (HV) then low valence (LV) order of environmental stimuli is presented in Fig. S1.

1.1 Figures

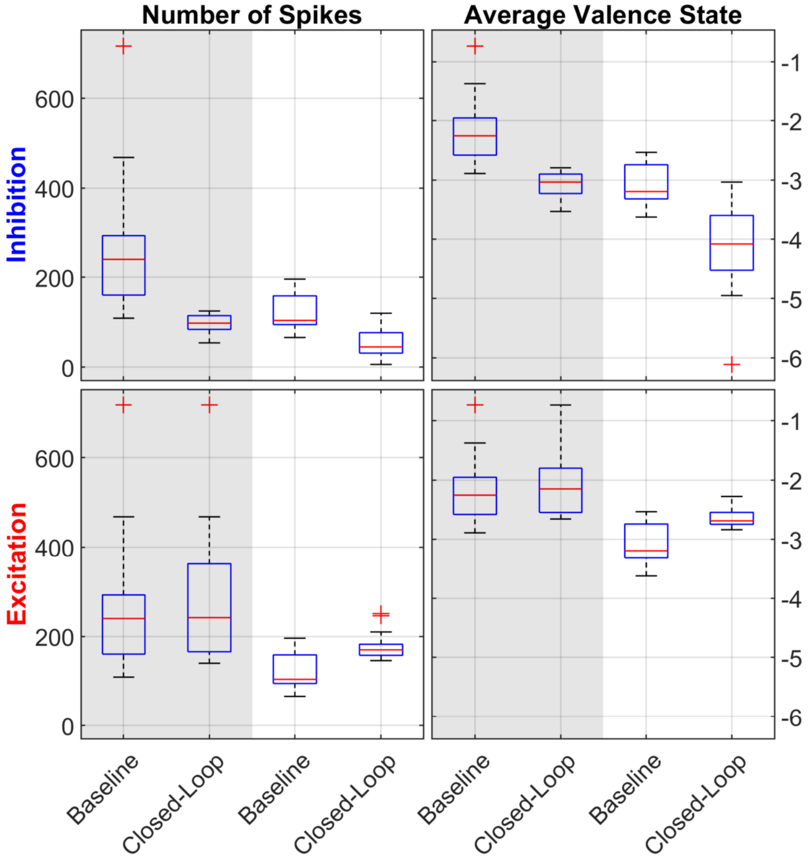

Figure S1. Statistical analysis with boxplot (N=17) visualization of HV then LV environmental stimuli order. The left column of sub-panels show the number of spikes in a given period, while the right column of sub-panels depict the average valence state. The top row of sub-panels show results from the inhibitory controller and the bottom one for the excitatory one. Within each sub-plot, the white background depicts LV periods while the gray-shaded areas show HV results. Each pair of data (i.e. baseline and closed-loop) was used during the *t*-tests analysis. Comparing the open-loop baseline and closed-loop results of number of spikes and average valence levels, LV periods are statistically significant both in inhibition and excitation (all sub-panels, white background). For HV periods, results are statistically significant only for inhibition (top-row, gray background).

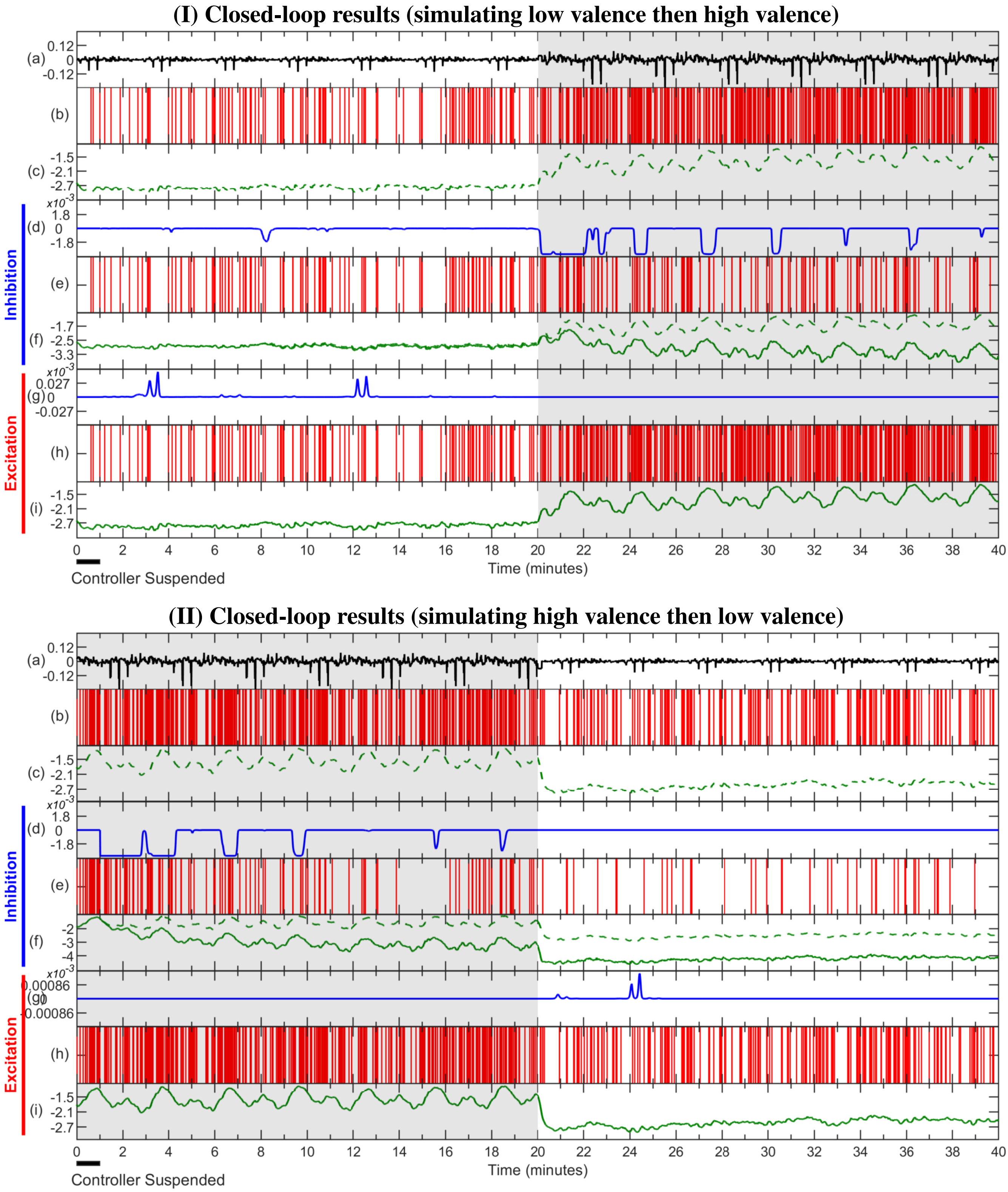

Figure S2. Simulation results of open-loop, inhibitory closed-loop and excitatory closed-loop scenarios for subject 1. In sub-figure I the external stimulus is comprised of half LV, then half HV, with sub-figure II being the opposite. In both I and II, LV and HV periods are represented with unshaded and grey-shaded areas, respectively. Sub-panel (a) depicts environmental stimulus (black) used in all three simulation scenarios. The sub-panels (b) and (c) show spike activity (red) and estimated valence state (green, dashed) during the open-loop, respectively. Sub-panels (d, e, f) display inhibitory closed-loop results, with (d) showing control effort (blue), (e) the corresponding binary signal (red) and (f) the comparison between open-loop (green, dashed) and closed-loop (green, solid) valence state. In a similar fashion, sub-panels (g, h, i) exhibit the excitatory closed-loop outcome.

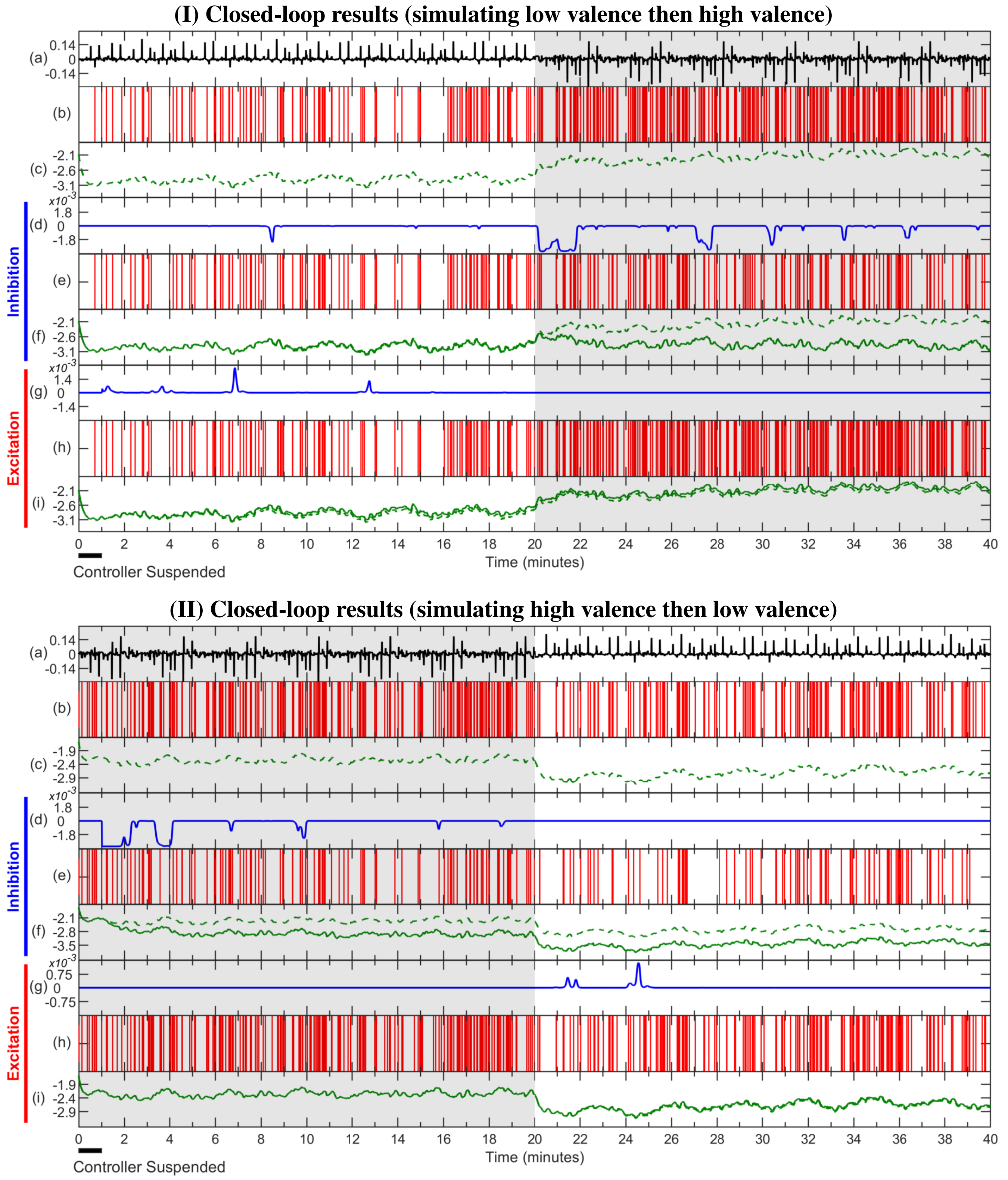

Figure S3. Simulation results of open-loop, inhibitory closed-loop and excitatory closed-loop scenarios for subject 2. In sub-figure I the external stimulus is comprised of half LV, then half HV, with sub-figure II being the opposite. In both I and II, LV and HV periods are represented with unshaded and grey-shaded areas, respectively. Sub-panel (a) depicts environmental stimulus (black) used in all three simulation scenarios. The sub-panels (b) and (c) show spike activity (red) and estimated valence state (green, dashed) during the open-loop, respectively. Sub-panels (d, e, f) display inhibitory closed-loop results, with (d) showing control effort (blue), (e) the corresponding binary signal (red) and (f) the comparison between open-loop (green, dashed) and closed-loop (green, solid) valence state. In a similar fashion, sub-panels (g, h, i) exhibit the excitatory closed-loop outcome.

(I) Closed-loop results (simulating low valence then high valence)

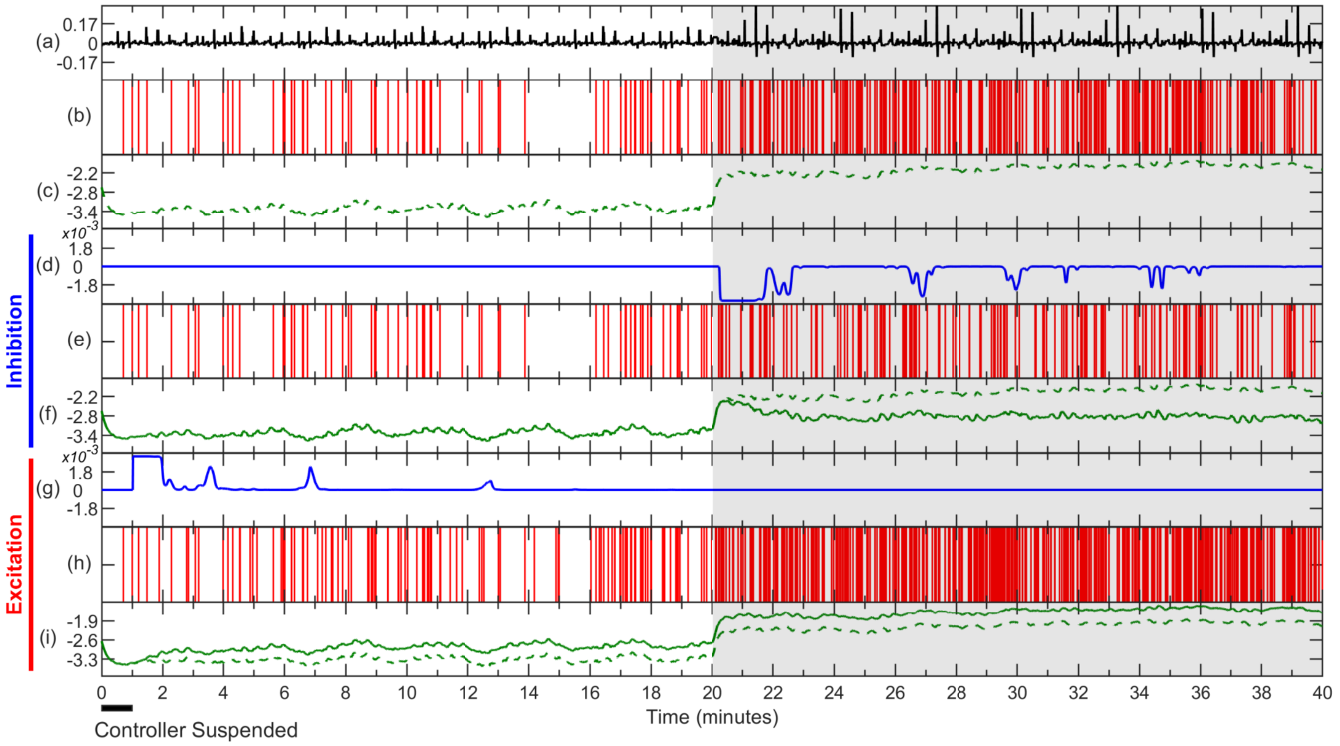

(II) Closed-loop results (simulating high valence then low valence)

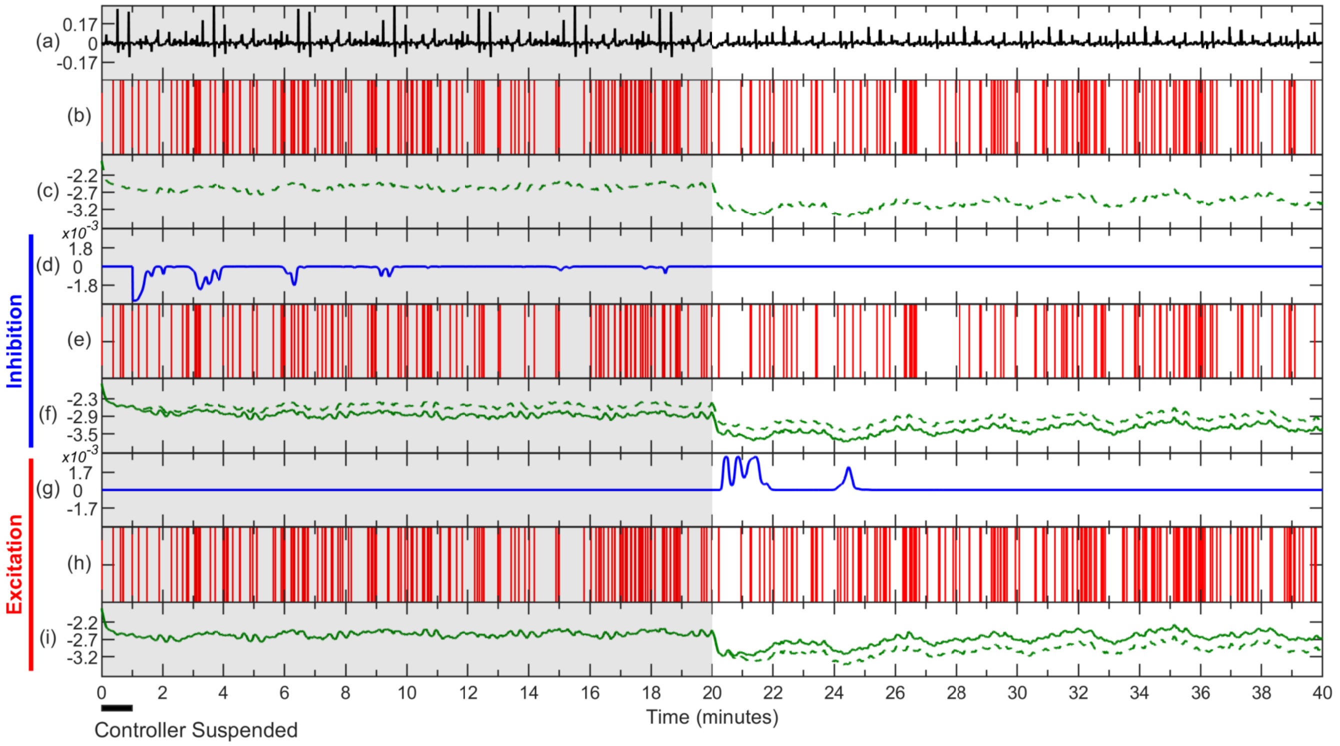

Figure S4. Simulation results of open-loop, inhibitory closed-loop and excitatory closed-loop scenarios for subject 3. In sub-figure I the external stimulus is comprised of half LV, then half HV, with sub-figure II being the opposite. In both I and II, LV and HV periods are represented with unshaded and grey-shaded areas, respectively. Sub-panel (a) depicts environmental stimulus (black) used in all three simulation scenarios. The sub-panels (b) and (c) show spike activity (red) and estimated valence state (green, dashed) during the open-loop, respectively. Sub-panels (d, e, f) display inhibitory closed-loop results, with (d) showing control effort (blue), (e) the corresponding binary signal (red) and (f) the comparison between open-loop (green, dashed) and closed-loop (green, solid) valence state. In a similar fashion, sub-panels (g, h, i) exhibit the excitatory closed-loop outcome.

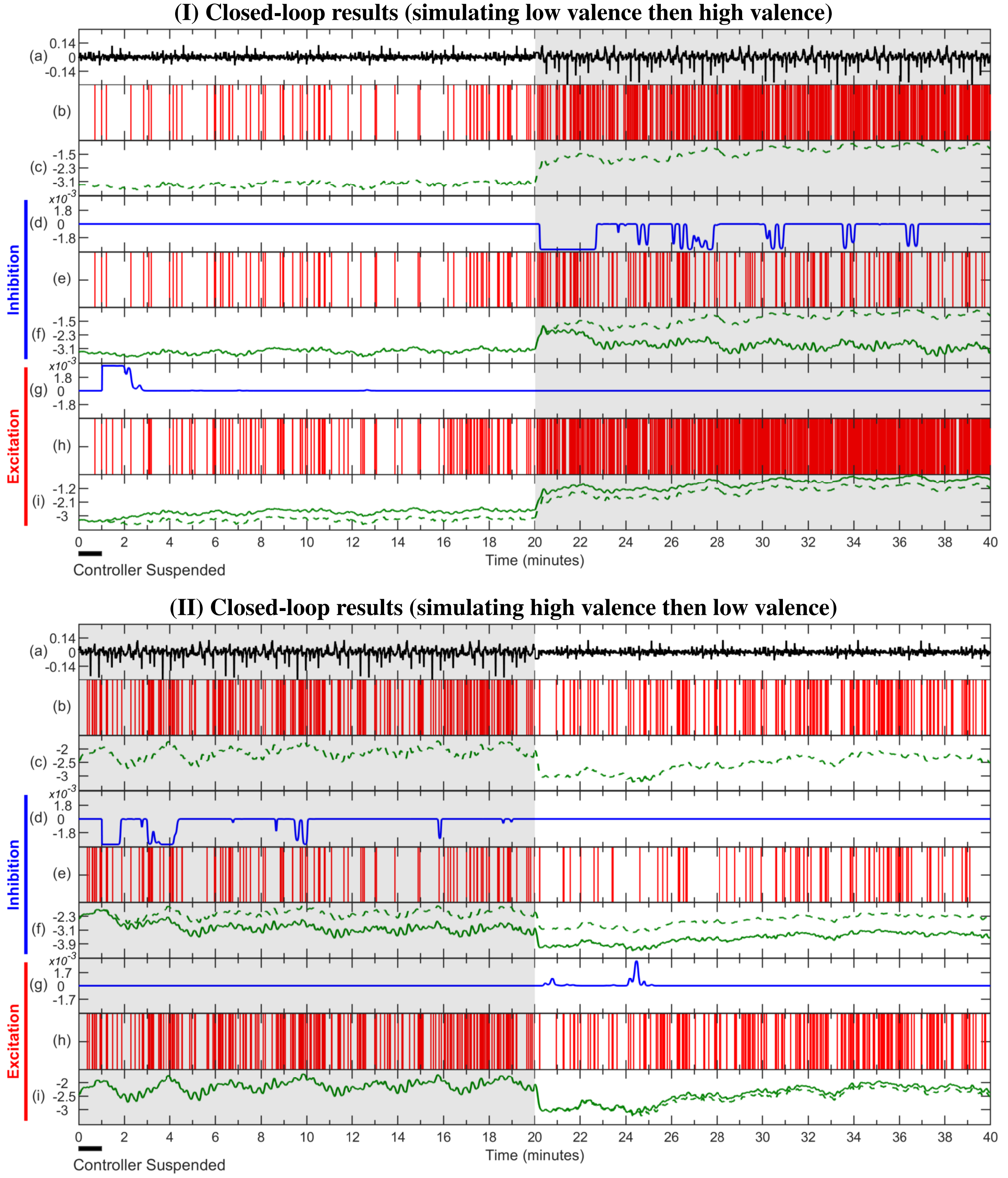

Figure S5. Simulation results of open-loop, inhibitory closed-loop and excitatory closed-loop scenarios for subject 5. In sub-figure I the external stimulus is comprised of half LV, then half HV, with sub-figure II being the opposite. In both I and II, LV and HV periods are represented with unshaded and grey-shaded areas, respectively. Sub-panel (a) depicts environmental stimulus (black) used in all three simulation scenarios. The sub-panels (b) and (c) show spike activity (red) and estimated valence state (green, dashed) during the open-loop, respectively. Sub-panels (d, e, f) display inhibitory closed-loop results, with (d) showing control effort (blue), (e) the corresponding binary signal (red) and (f) the comparison between open-loop (green, dashed) and closed-loop (green, solid) valence state. In a similar fashion, sub-panels (g, h, i) exhibit the excitatory closed-loop outcome.

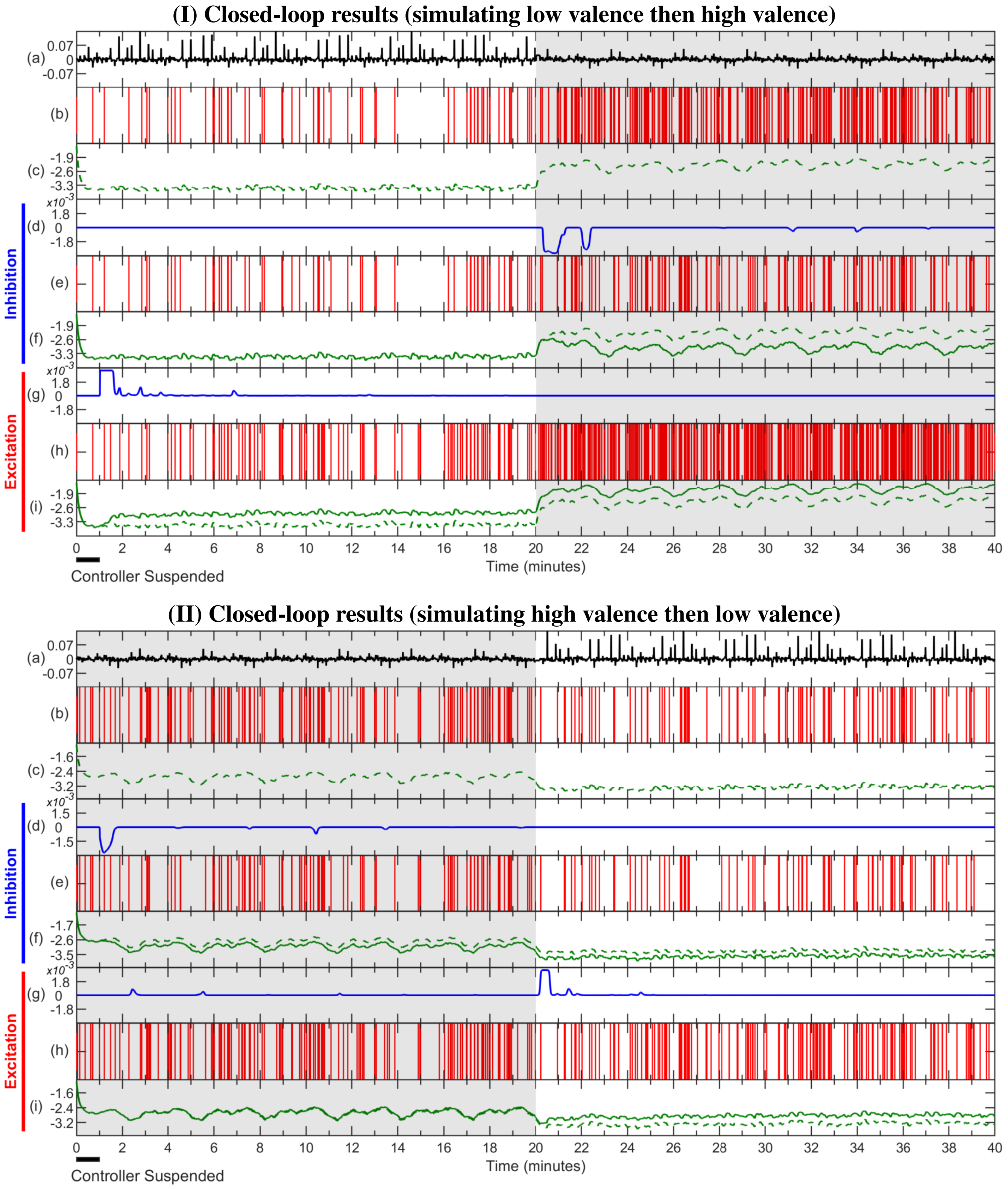

Figure S6. Simulation results of open-loop, inhibitory closed-loop and excitatory closed-loop scenarios for subject 6. In sub-figure I the external stimulus is comprised of half LV, then half HV, with sub-figure II being the opposite. In both I and II, LV and HV periods are represented with unshaded and grey-shaded areas, respectively. Sub-panel (a) depicts environmental stimulus (black) used in all three simulation scenarios. The sub-panels (b) and (c) show spike activity (red) and estimated valence state (green, dashed) during the open-loop, respectively. Sub-panels (d, e, f) display inhibitory closed-loop results, with (d) showing control effort (blue), (e) the corresponding binary signal (red) and (f) the comparison between open-loop (green, dashed) and closed-loop (green, solid) valence state. In a similar fashion, sub-panels (g, h, i) exhibit the excitatory closed-loop outcome.

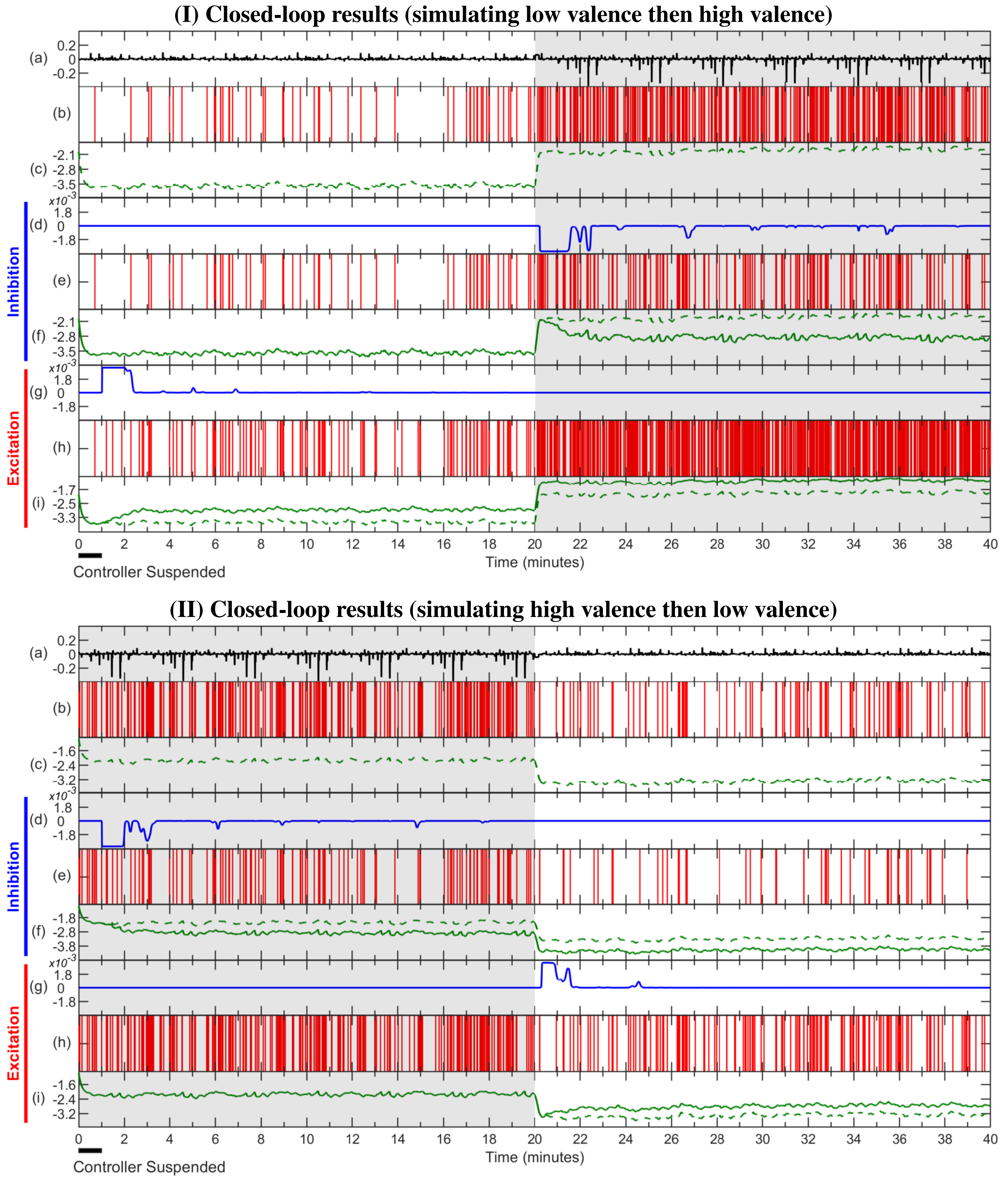

Figure S7. Simulation results of open-loop, inhibitory closed-loop and excitatory closed-loop scenarios for subject 7. In sub-figure I the external stimulus is comprised of half LV, then half HV, with sub-figure II being the opposite. In both I and II, LV and HV periods are represented with unshaded and grey-shaded areas, respectively. Sub-panel (a) depicts environmental stimulus (black) used in all three simulation scenarios. The sub-panels (b) and (c) show spike activity (red) and estimated valence state (green, dashed) during the open-loop, respectively. Sub-panels (d, e, f) display inhibitory closed-loop results, with (d) showing control effort (blue), (e) the corresponding binary signal (red) and (f) the comparison between open-loop (green, dashed) and closed-loop (green, solid) valence state. In a similar fashion, sub-panels (g, h, i) exhibit the excitatory closed-loop outcome.

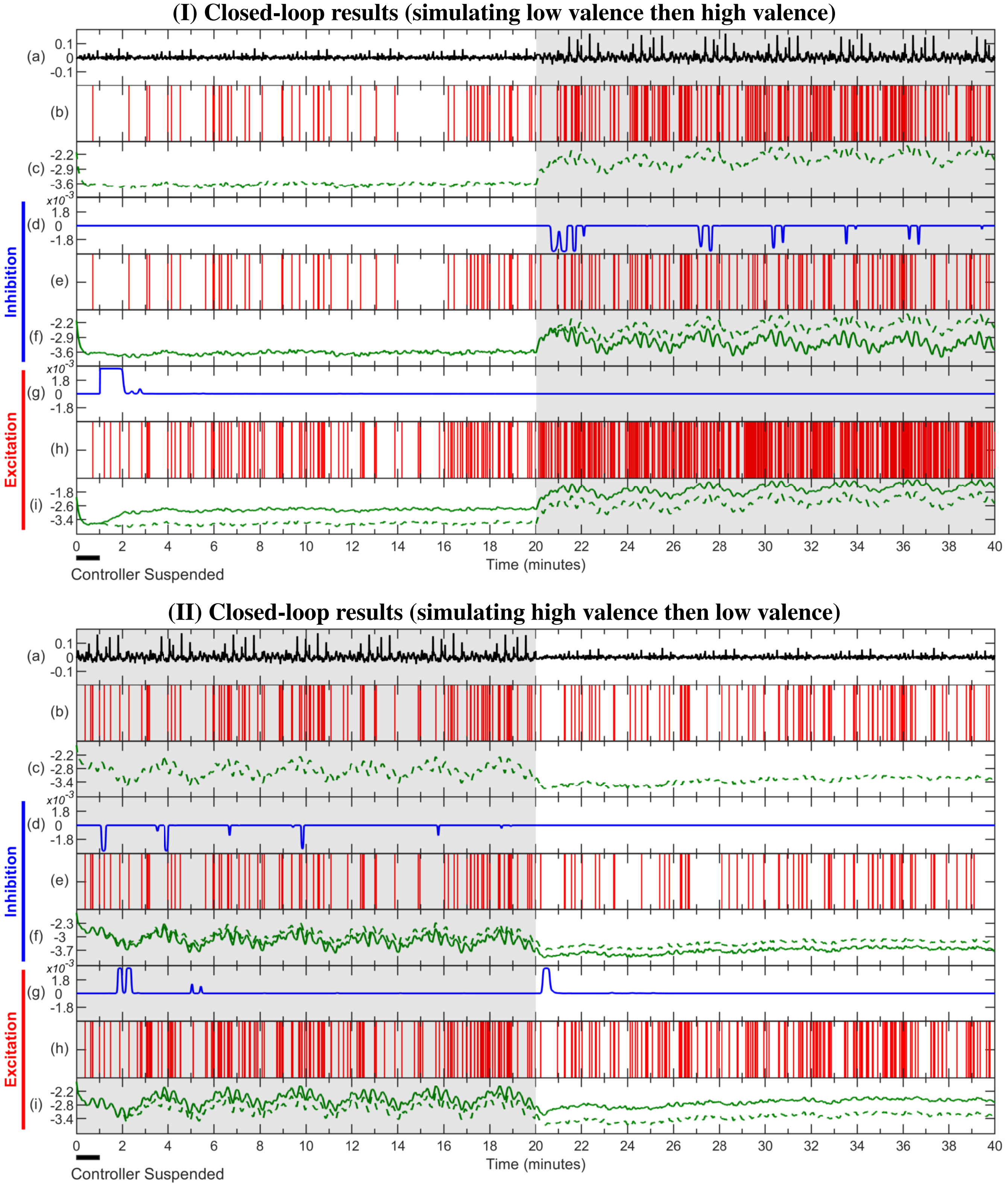

Figure S8. Simulation results of open-loop, inhibitory closed-loop and excitatory closed-loop scenarios for subject 8. In sub-figure I the external stimulus is comprised of half LV, then half HV, with sub-figure II being the opposite. In both I and II, LV and HV periods are represented with unshaded and grey-shaded areas, respectively. Sub-panel (a) depicts environmental stimulus (black) used in all three simulation scenarios. The sub-panels (b) and (c) show spike activity (red) and estimated valence state (green, dashed) during the open-loop, respectively. Sub-panels (d, e, f) display inhibitory closed-loop results, with (d) showing control effort (blue), (e) the corresponding binary signal (red) and (f) the comparison between open-loop (green, dashed) and closed-loop (green, solid) valence state. In a similar fashion, sub-panels (g, h, i) exhibit the excitatory closed-loop outcome.

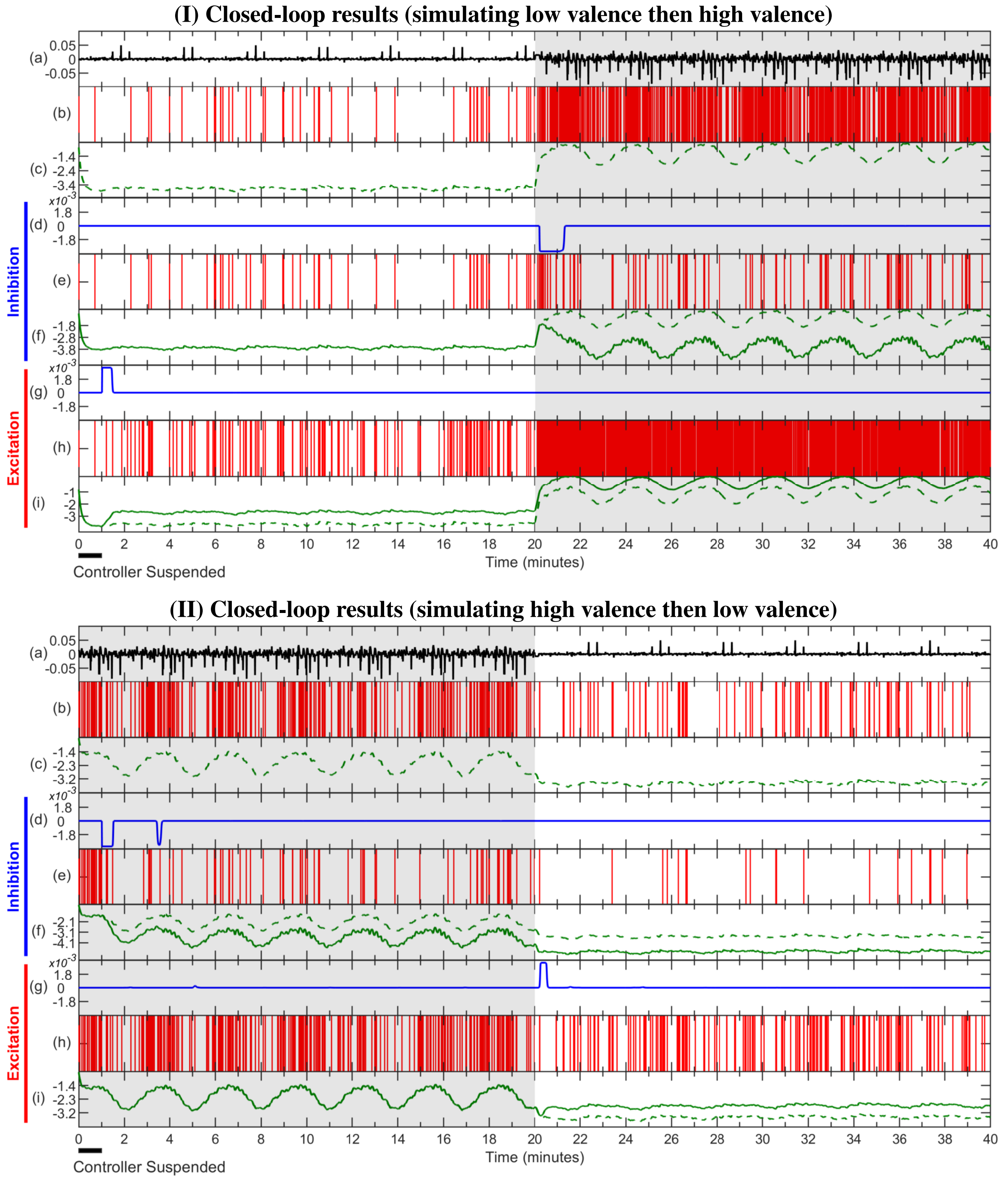

Figure S9. Simulation results of open-loop, inhibitory closed-loop and excitatory closed-loop scenarios for subject 9. In sub-figure I the external stimulus is comprised of half LV, then half HV, with sub-figure II being the opposite. In both I and II, LV and HV periods are represented with unshaded and grey-shaded areas, respectively. Sub-panel (a) depicts environmental stimulus (black) used in all three simulation scenarios. The sub-panels (b) and (c) show spike activity (red) and estimated valence state (green, dashed) during the open-loop, respectively. Sub-panels (d, e, f) display inhibitory closed-loop results, with (d) showing control effort (blue), (e) the corresponding binary signal (red) and (f) the comparison between open-loop (green, dashed) and closed-loop (green, solid) valence state. In a similar fashion, sub-panels (g, h, i) exhibit the excitatory closed-loop outcome.

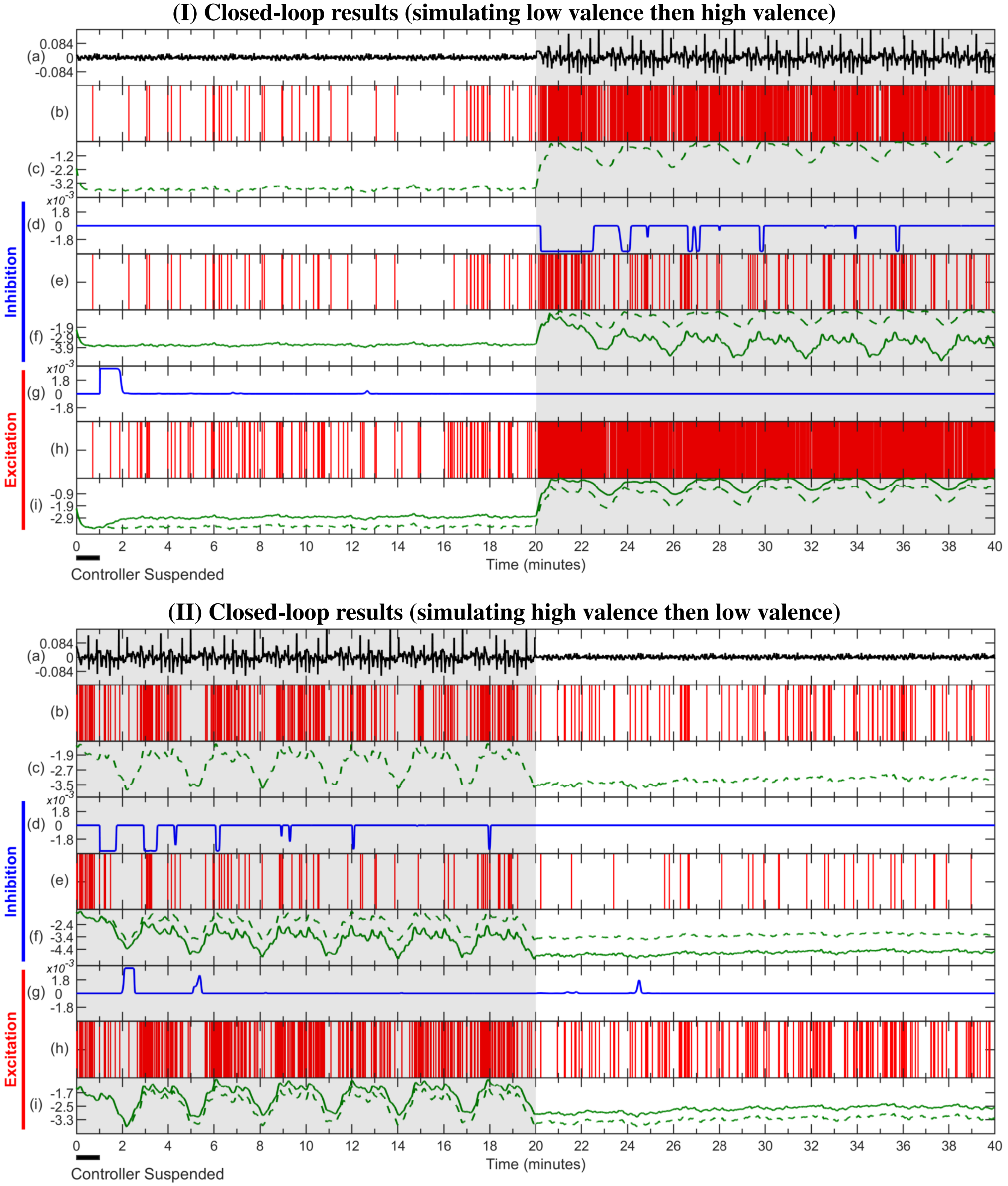

Figure S10. Simulation results of open-loop, inhibitory closed-loop and excitatory closed-loop scenarios for subject 10. In sub-figure I the external stimulus is comprised of half LV, then half HV, with sub-figure II being the opposite. In both I and II, LV and HV periods are represented with unshaded and grey-shaded areas, respectively. Sub-panel (a) depicts environmental stimulus (black) used in all three simulation scenarios. The sub-panels (b) and (c) show spike activity (red) and estimated valence state (green, dashed) during the open-loop, respectively. Sub-panels (d, e, f) display inhibitory closed-loop results, with (d) showing control effort (blue), (e) the corresponding binary signal (red) and (f) the comparison between open-loop (green, dashed) and closed-loop (green, solid) valence state. In a similar fashion, sub-panels (g, h, i) exhibit the excitatory closed-loop outcome.

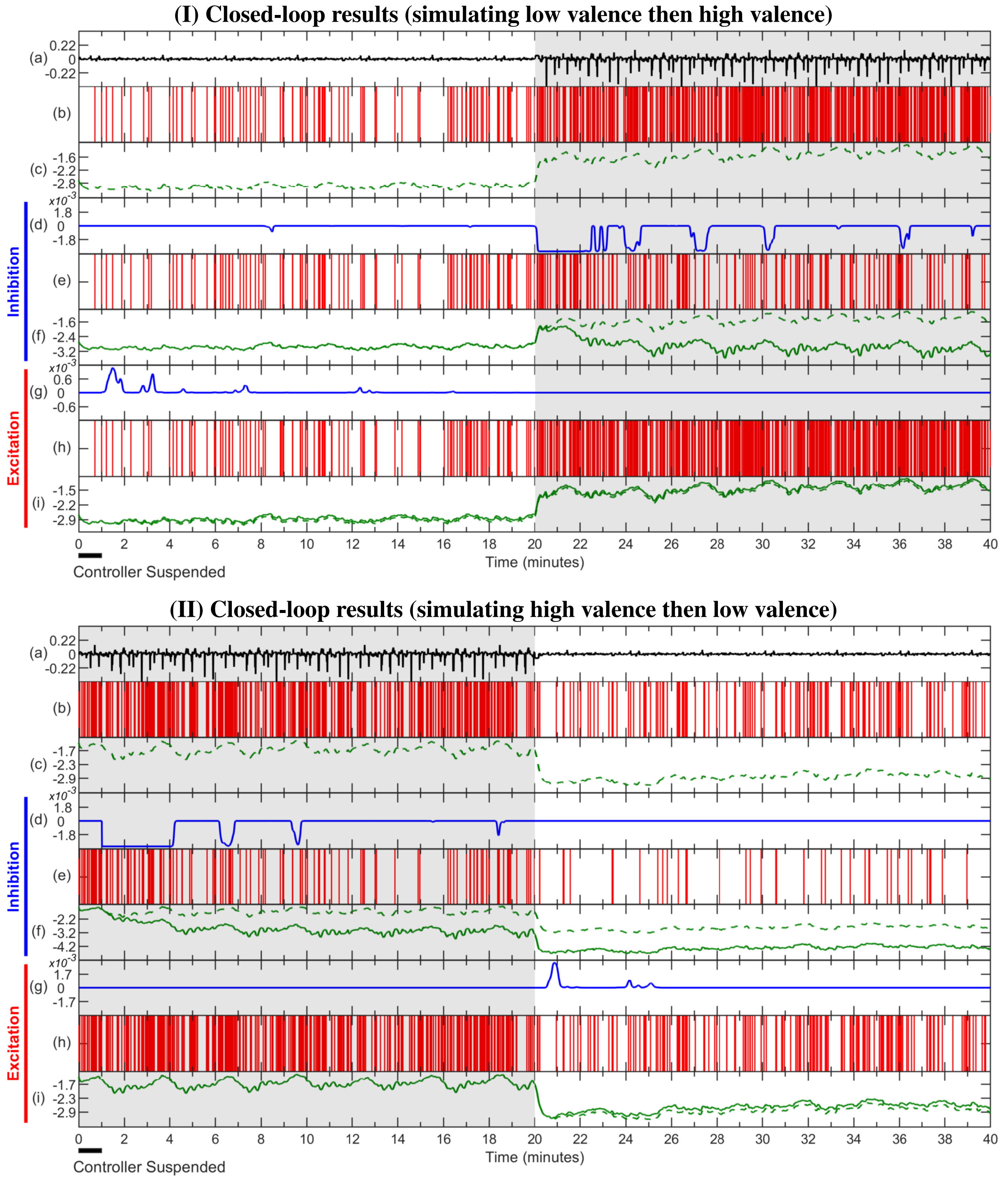

Figure S11. Simulation results of open-loop, inhibitory closed-loop and excitatory closed-loop scenarios for subject 13. In sub-figure I the external stimulus is comprised of half LV, then half HV, with sub-figure II being the opposite. In both I and II, LV and HV periods are represented with unshaded and grey-shaded areas, respectively. Sub-panel (a) depicts environmental stimulus (black) used in all three simulation scenarios. The sub-panels (b) and (c) show spike activity (red) and estimated valence state (green, dashed) during the open-loop, respectively. Sub-panels (d, e, f) display inhibitory closed-loop results, with (d) showing control effort (blue), (e) the corresponding binary signal (red) and (f) the comparison between open-loop (green, dashed) and closed-loop (green, solid) valence state. In a similar fashion, sub-panels (g, h, i) exhibit the excitatory closed-loop outcome.

(I) Closed-loop results (simulating low valence then high valence)

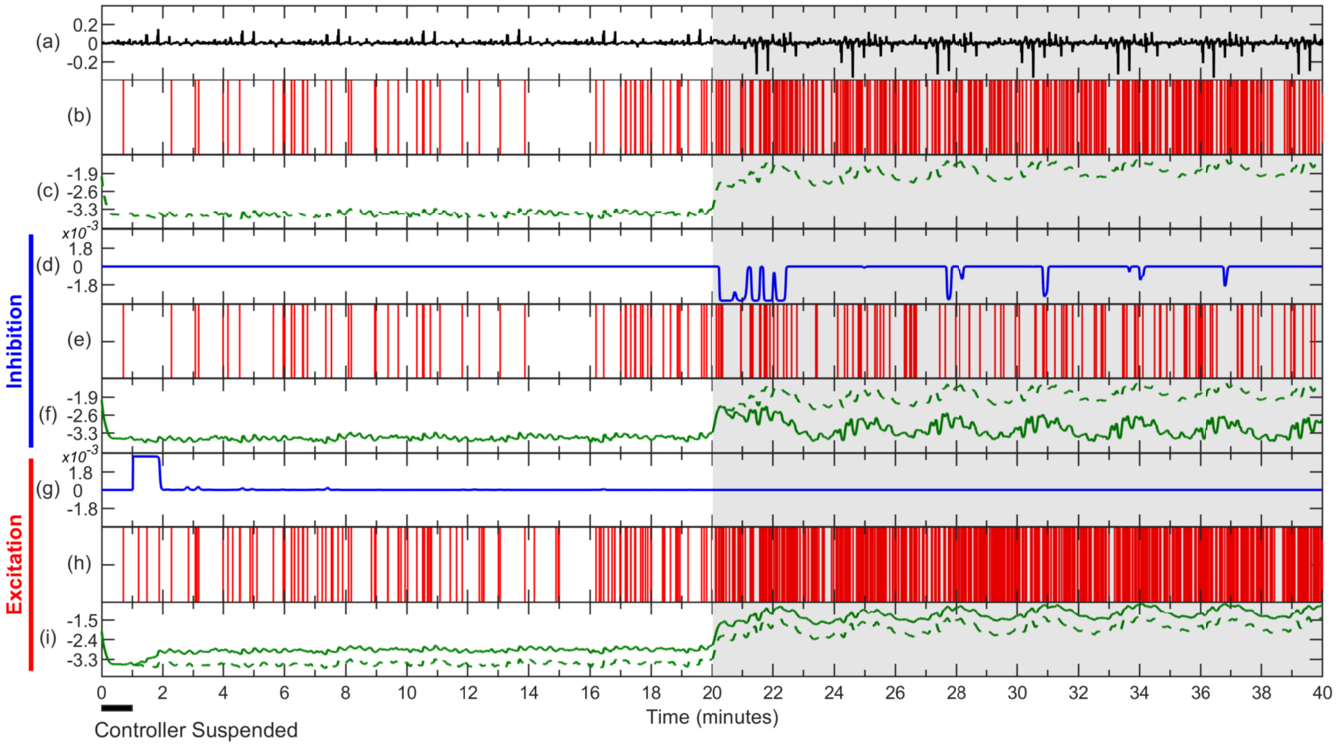

(II) Closed-loop results (simulating high valence then low valence)

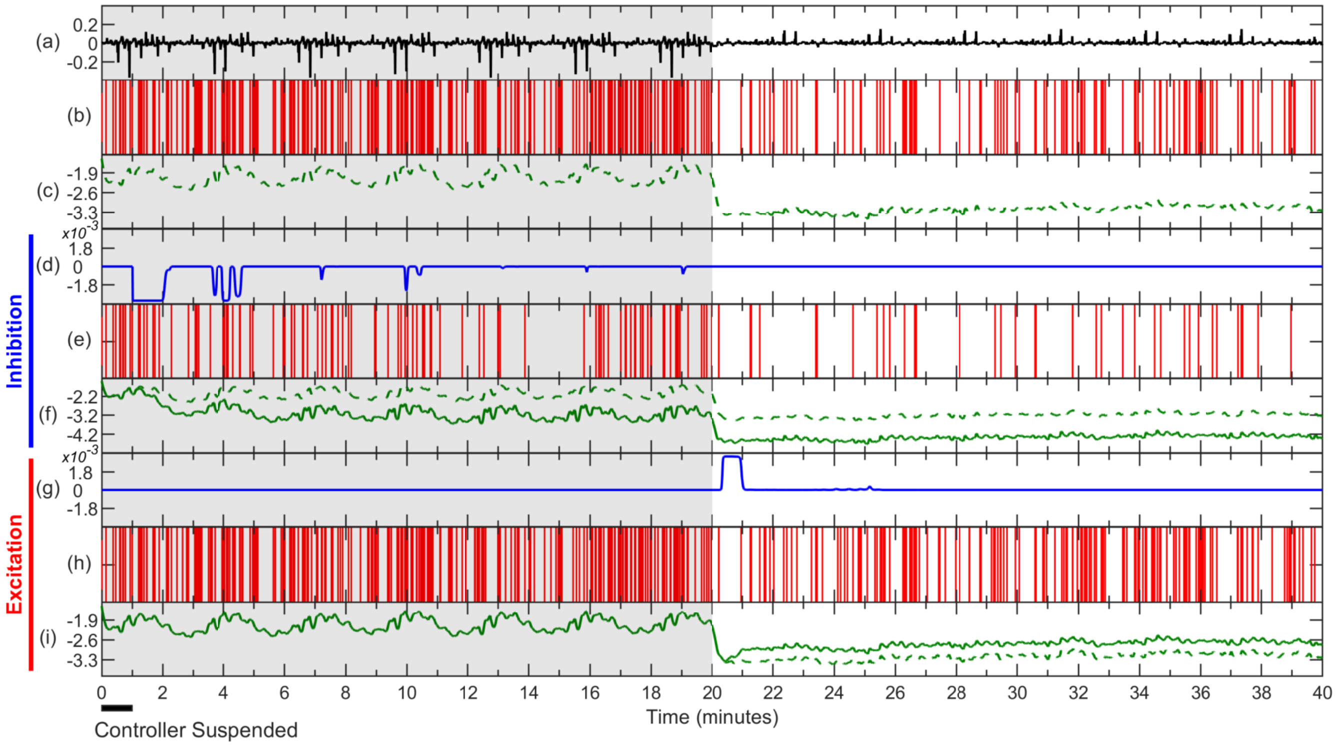

Figure S12. Simulation results of open-loop, inhibitory closed-loop and excitatory closed-loop scenarios for subject 14. In sub-figure I the external stimulus is comprised of half LV, then half HV, with sub-figure II being the opposite. In both I and II, LV and HV periods are represented with unshaded and grey-shaded areas, respectively. Sub-panel (a) depicts environmental stimulus (black) used in all three simulation scenarios. The sub-panels (b) and (c) show spike activity (red) and estimated valence state (green, dashed) during the open-loop, respectively. Sub-panels (d, e, f) display inhibitory closed-loop results, with (d) showing control effort (blue), (e) the corresponding binary signal (red) and (f) the comparison between open-loop (green, dashed) and closed-loop (green, solid) valence state. In a similar fashion, sub-panels (g, h, i) exhibit the excitatory closed-loop outcome.

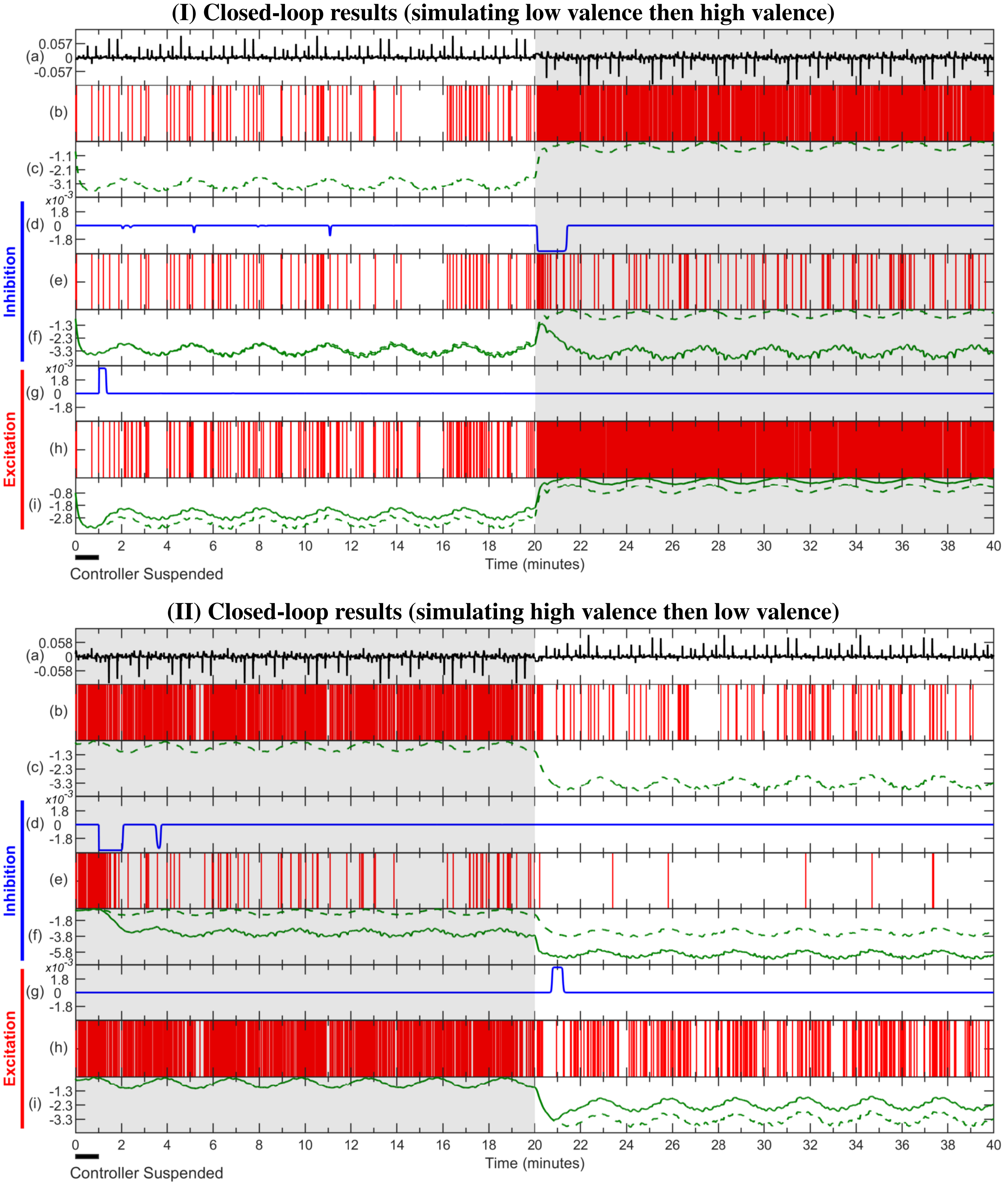

Figure S13. Simulation results of open-loop, inhibitory closed-loop and excitatory closed-loop scenarios for subject 15. In sub-figure I the external stimulus is comprised of half LV, then half HV, with sub-figure II being the opposite. In both I and II, LV and HV periods are represented with unshaded and grey-shaded areas, respectively. Sub-panel (a) depicts environmental stimulus (black) used in all three simulation scenarios. The sub-panels (b) and (c) show spike activity (red) and estimated valence state (green, dashed) during the open-loop, respectively. Sub-panels (d, e, f) display inhibitory closed-loop results, with (d) showing control effort (blue), (e) the corresponding binary signal (red) and (f) the comparison between open-loop (green, dashed) and closed-loop (green, solid) valence state. In a similar fashion, sub-panels (g, h, i) exhibit the excitatory closed-loop outcome.

(I) Closed-loop results (simulating low valence then high valence)

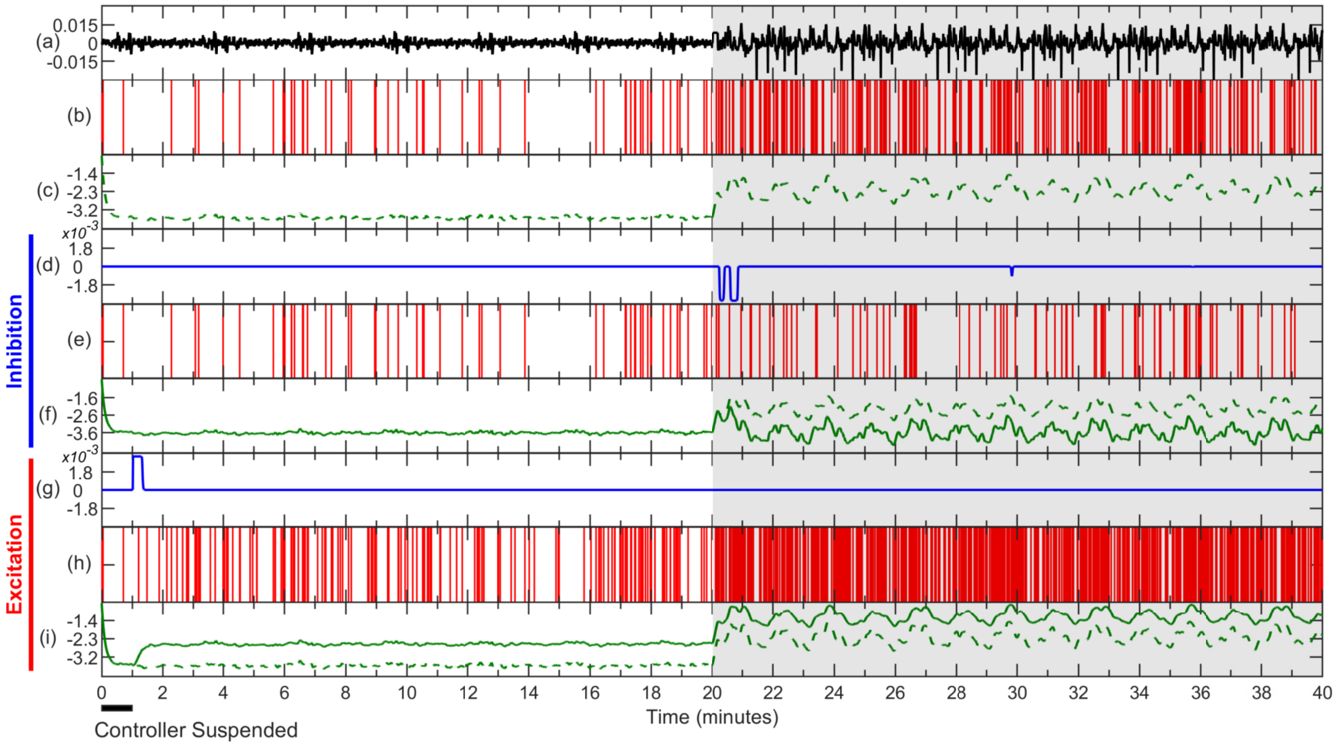

(II) Closed-loop results (simulating high valence then low valence)

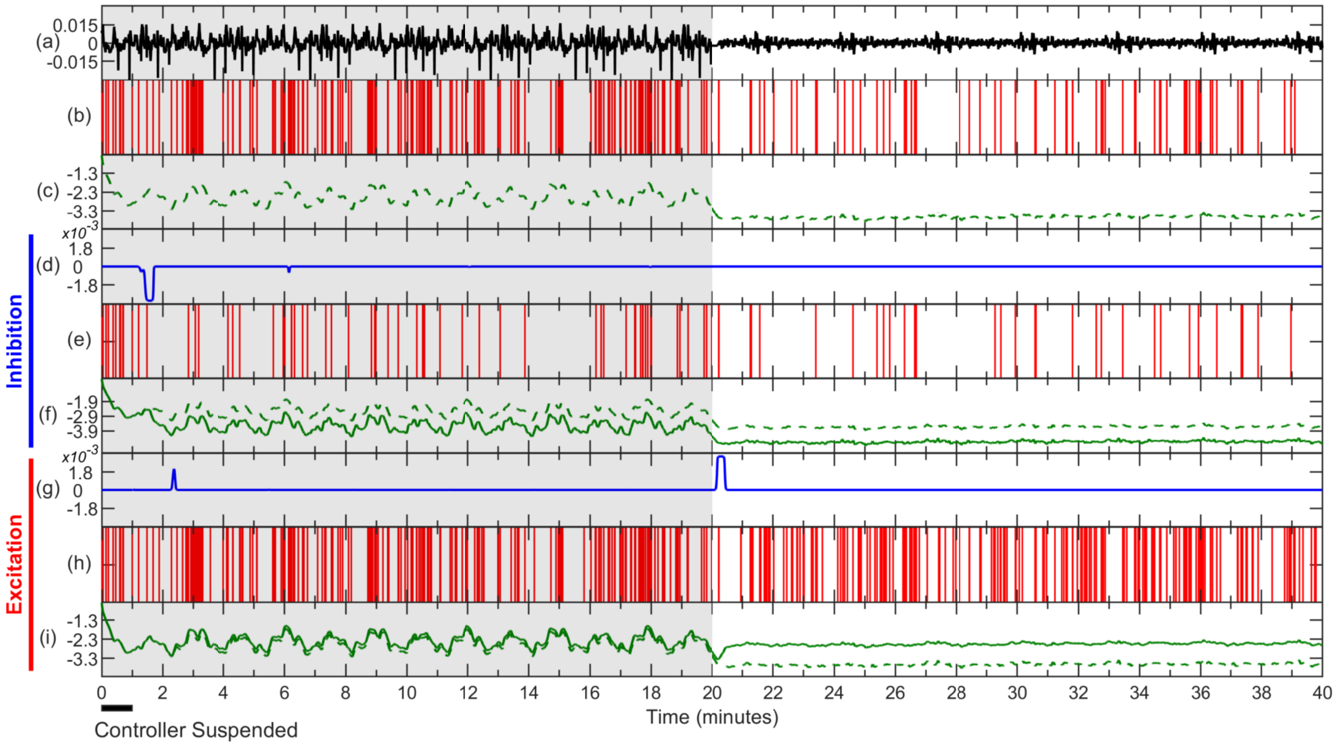

Figure S14. Simulation results of open-loop, inhibitory closed-loop and excitatory closed-loop scenarios for subject 16. In sub-figure I the external stimulus is comprised of half LV, then half HV, with sub-figure II being the opposite. In both I and II, LV and HV periods are represented with unshaded and grey-shaded areas, respectively. Sub-panel (a) depicts environmental stimulus (black) used in all three simulation scenarios. The sub-panels (b) and (c) show spike activity (red) and estimated valence state (green, dashed) during the open-loop, respectively. Sub-panels (d, e, f) display inhibitory closed-loop results, with (d) showing control effort (blue), (e) the corresponding binary signal (red) and (f) the comparison between open-loop (green, dashed) and closed-loop (green, solid) valence state. In a similar fashion, sub-panels (g, h, i) exhibit the excitatory closed-loop outcome.

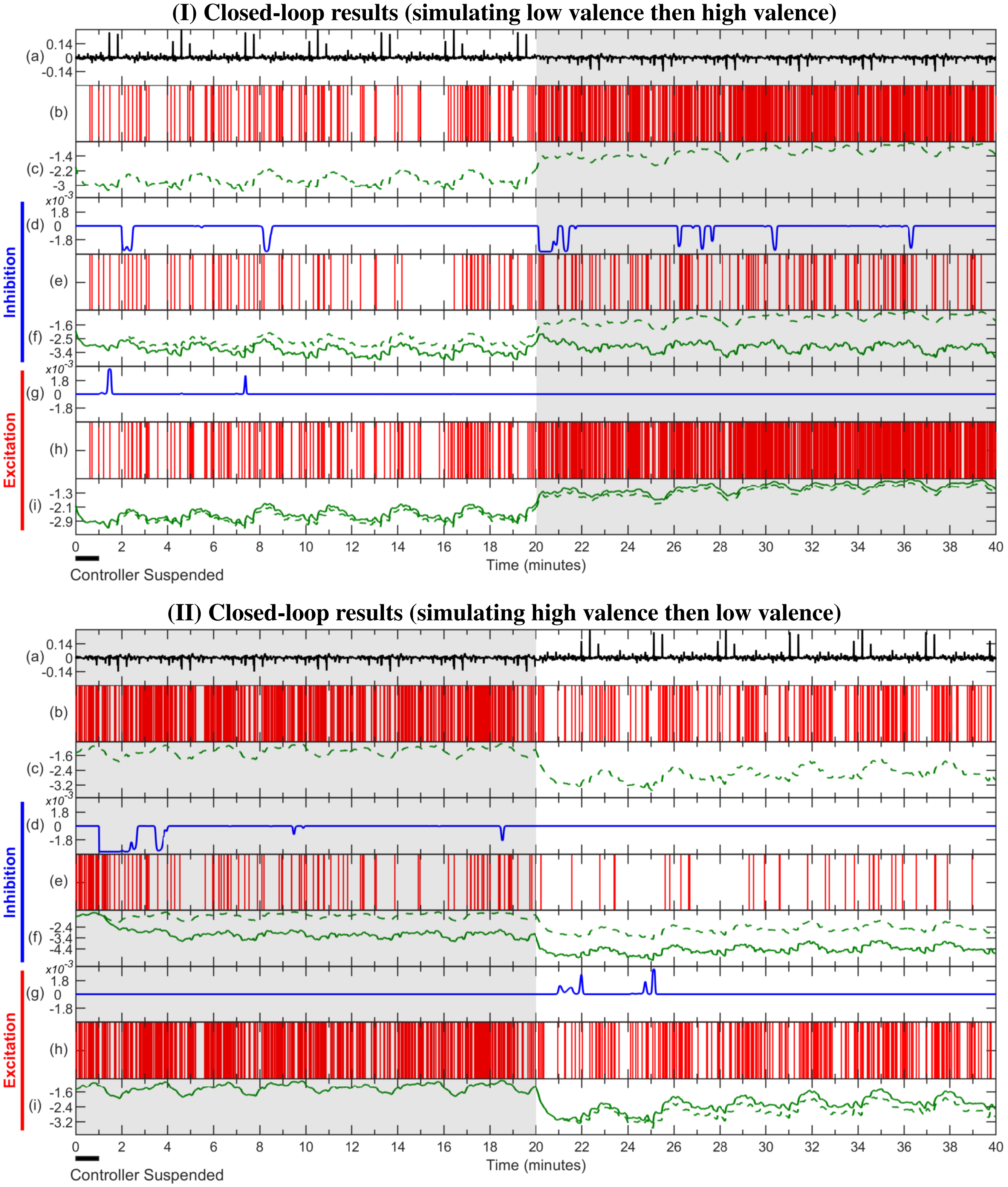

Figure S15. Simulation results of open-loop, inhibitory closed-loop and excitatory closed-loop scenarios for subject 17. In sub-figure I the external stimulus is comprised of half LV, then half HV, with sub-figure II being the opposite. In both I and II, LV and HV periods are represented with unshaded and grey-shaded areas, respectively. Sub-panel (a) depicts environmental stimulus (black) used in all three simulation scenarios. The sub-panels (b) and (c) show spike activity (red) and estimated valence state (green, dashed) during the open-loop, respectively. Sub-panels (d, e, f) display inhibitory closed-loop results, with (d) showing control effort (blue), (e) the corresponding binary signal (red) and (f) the comparison between open-loop (green, dashed) and closed-loop (green, solid) valence state. In a similar fashion, sub-panels (g, h, i) exhibit the excitatory closed-loop outcome.

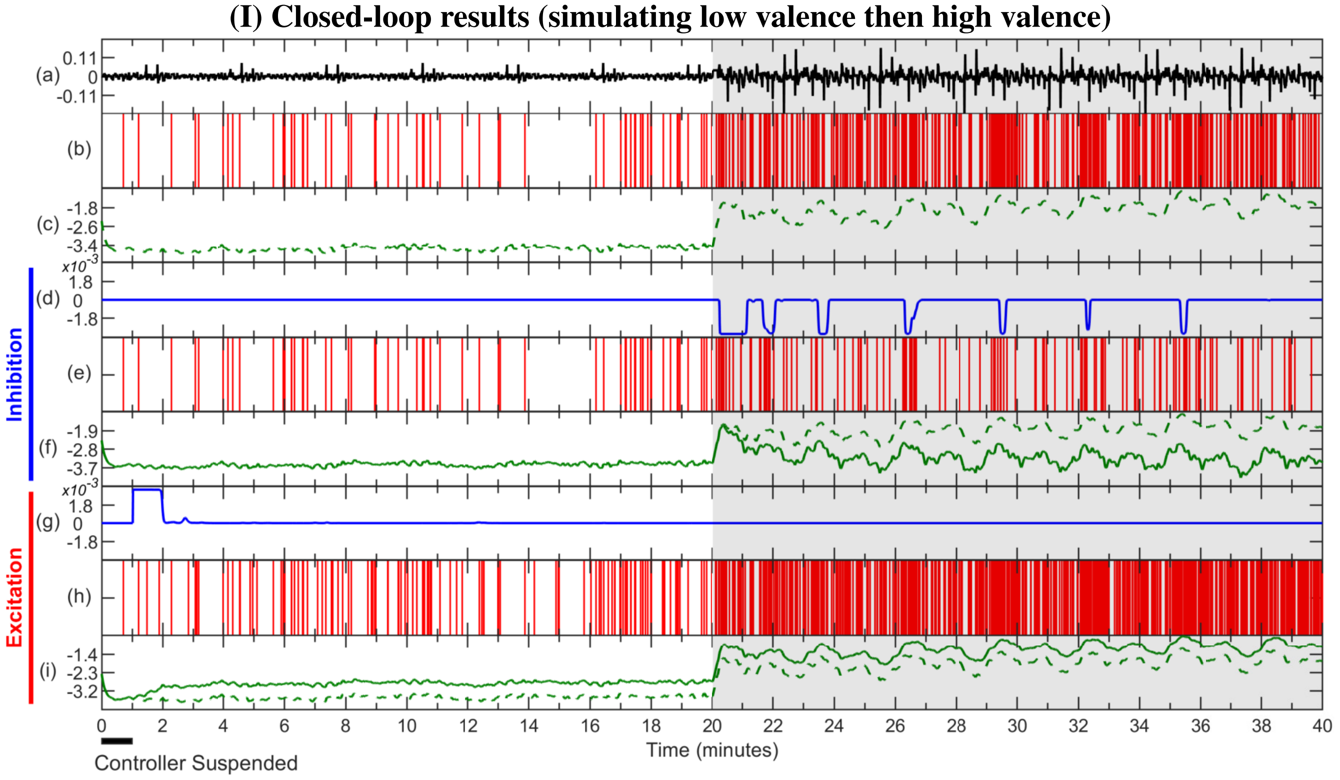

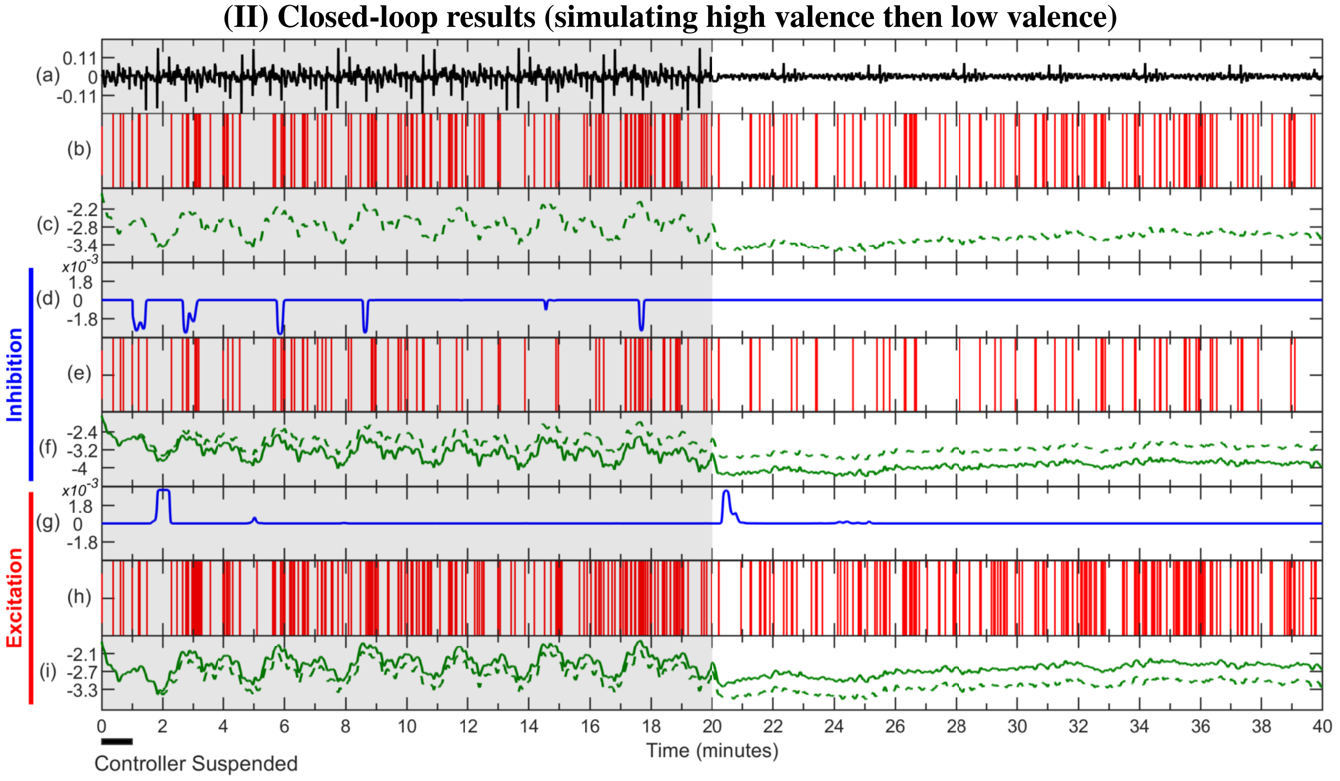

Figure S16. Simulation results of open-loop, inhibitory closed-loop and excitatory closed-loop scenarios for subject 18. In sub-figure I the external stimulus is comprised of half LV, then half HV, with sub-figure II being the opposite. In both I and II, LV and HV periods are represented with unshaded and grey-shaded areas, respectively. Sub-panel (a) depicts environmental stimulus (black) used in all three simulation scenarios. The sub-panels (b) and (c) show spike activity (red) and estimated valence state (green, dashed) during the open-loop, respectively. Sub-panels (d, e, f) display inhibitory closed-loop results, with (d) showing control effort (blue), (e) the corresponding binary signal (red) and (f) the comparison between open-loop (green, dashed) and closed-loop (green, solid) valence state. In a similar fashion, sub-panels (g, h, i) exhibit the excitatory closed-loop outcome.

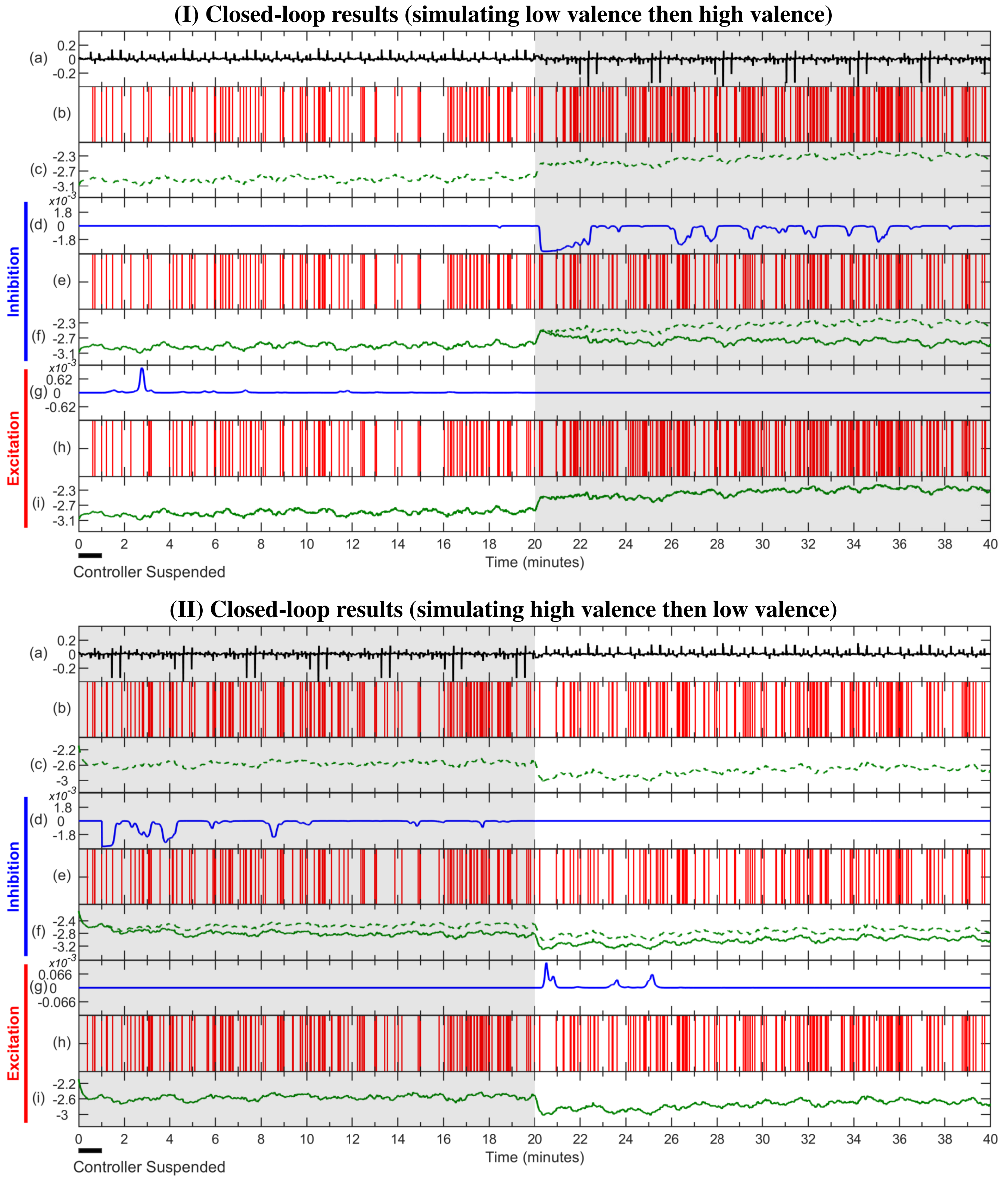

Figure S17. Simulation results of open-loop, inhibitory closed-loop and excitatory closed-loop scenarios for subject 19. In sub-figure I the external stimulus is comprised of half LV, then half HV, with sub-figure II being the opposite. In both I and II, LV and HV periods are represented with unshaded and grey-shaded areas, respectively. Sub-panel (a) depicts environmental stimulus (black) used in all three simulation scenarios. The sub-panels (b) and (c) show spike activity (red) and estimated valence state (green, dashed) during the open-loop, respectively. Sub-panels (d, e, f) display inhibitory closed-loop results, with (d) showing control effort (blue), (e) the corresponding binary signal (red) and (f) the comparison between open-loop (green, dashed) and closed-loop (green, solid) valence state. In a similar fashion, sub-panels (g, h, i) exhibit the excitatory closed-loop outcome.

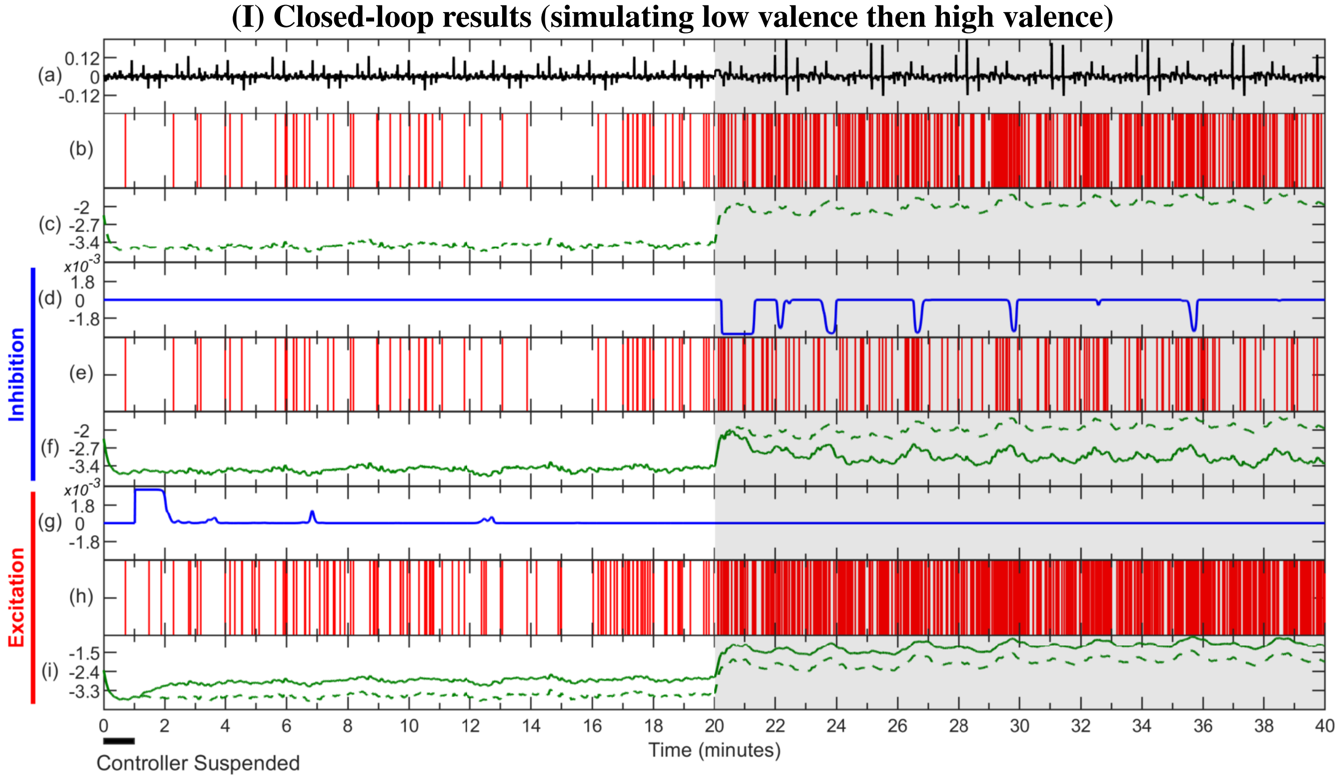

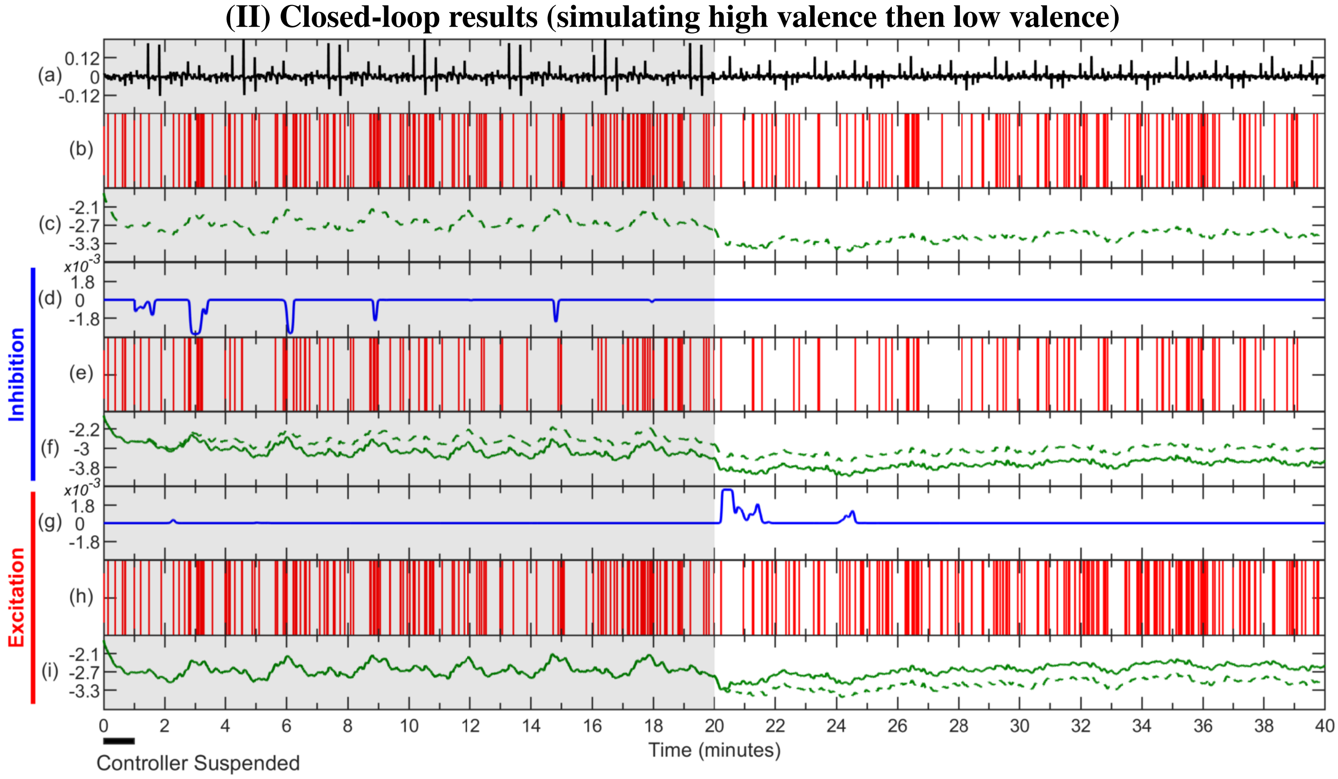

Figure S18. Simulation results of open-loop, inhibitory closed-loop and excitatory closed-loop scenarios for subject 22. In sub-figure I the external stimulus is comprised of half LV, then half HV, with sub-figure II being the opposite. In both I and II, LV and HV periods are represented with unshaded and grey-shaded areas, respectively. Sub-panel (a) depicts environmental stimulus (black) used in all three simulation scenarios. The sub-panels (b) and (c) show spike activity (red) and estimated valence state (green, dashed) during the open-loop, respectively. Sub-panels (d, e, f) display inhibitory closed-loop results, with (d) showing control effort (blue), (e) the corresponding binary signal (red) and (f) the comparison between open-loop (green, dashed) and closed-loop (green, solid) valence state. In a similar fashion, sub-panels (g, h, i) exhibit the excitatory closed-loop outcome.

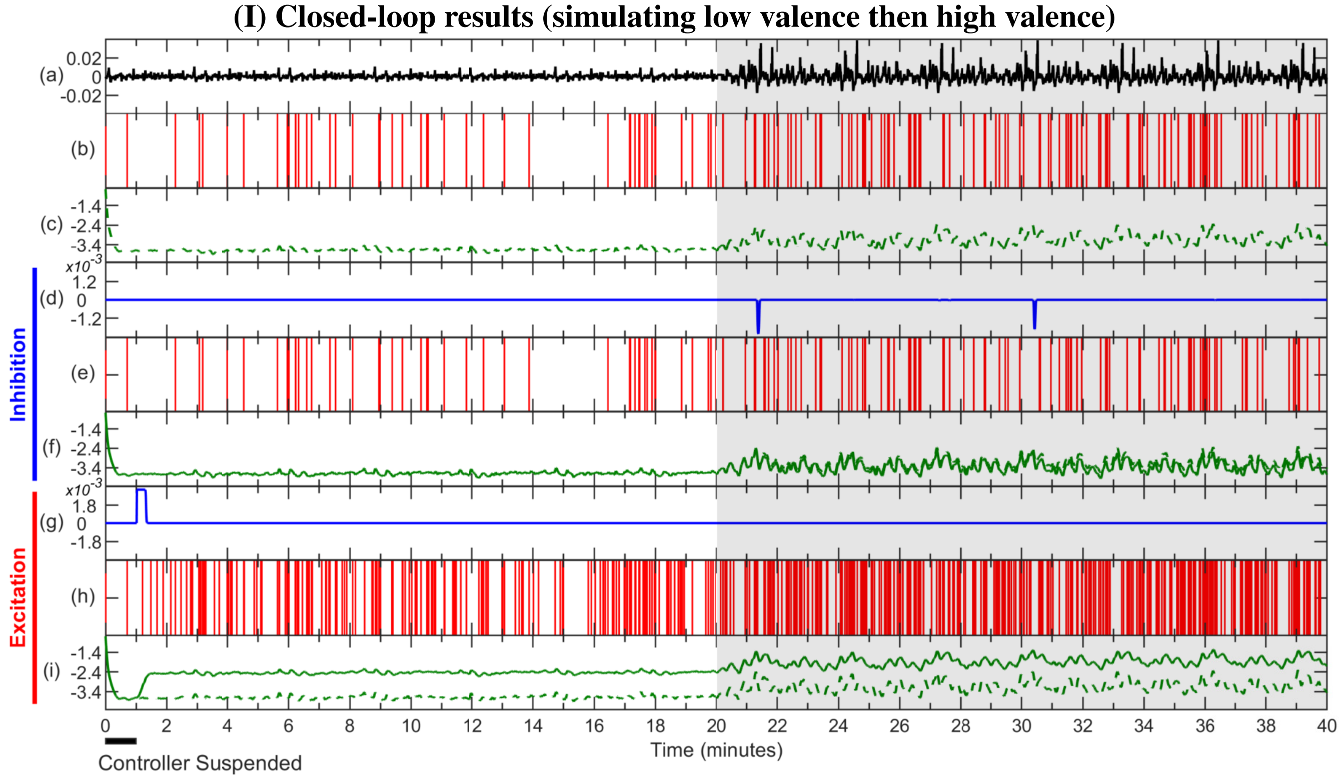

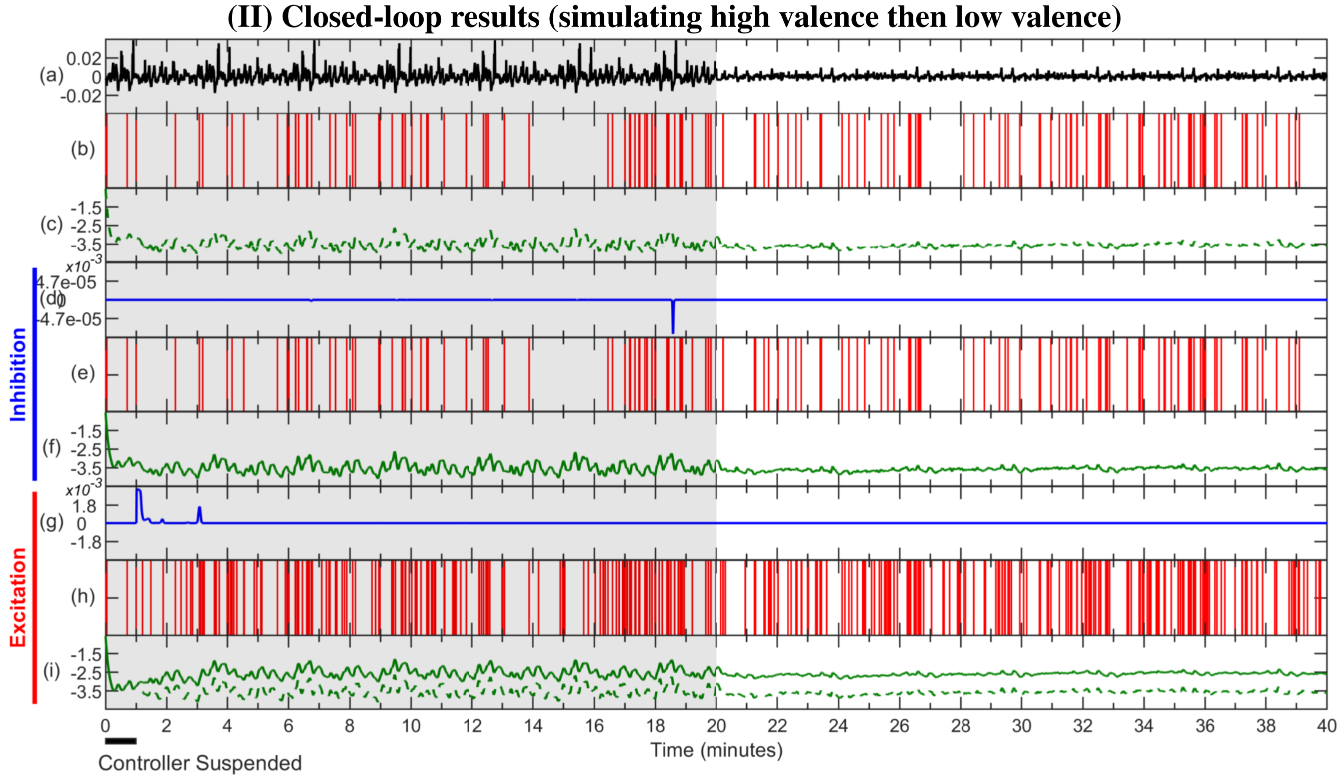

Figure S19. Simulation results of open-loop, inhibitory closed-loop and excitatory closed-loop scenarios for discarded subject 4. In sub-figure I the external stimulus is comprised of half LV, then half HV, with sub-figure II being the opposite. In both I and II, LV and HV periods are represented with unshaded and grey-shaded areas, respectively. Sub-panel (a) depicts environmental stimulus (black) used in all three simulation scenarios. The sub-panels (b) and (c) show spike activity (red) and estimated valence state (green, dashed) during the open-loop, respectively. Sub-panels (d, e, f) display inhibitory closed-loop results, with (d) showing control effort (blue), (e) the corresponding binary signal (red) and (f) the comparison between open-loop (green, dashed) and closed-loop (green, solid) valence state. In a similar fashion, sub-panels (g, h, i) exhibit the excitatory closed-loop outcome.

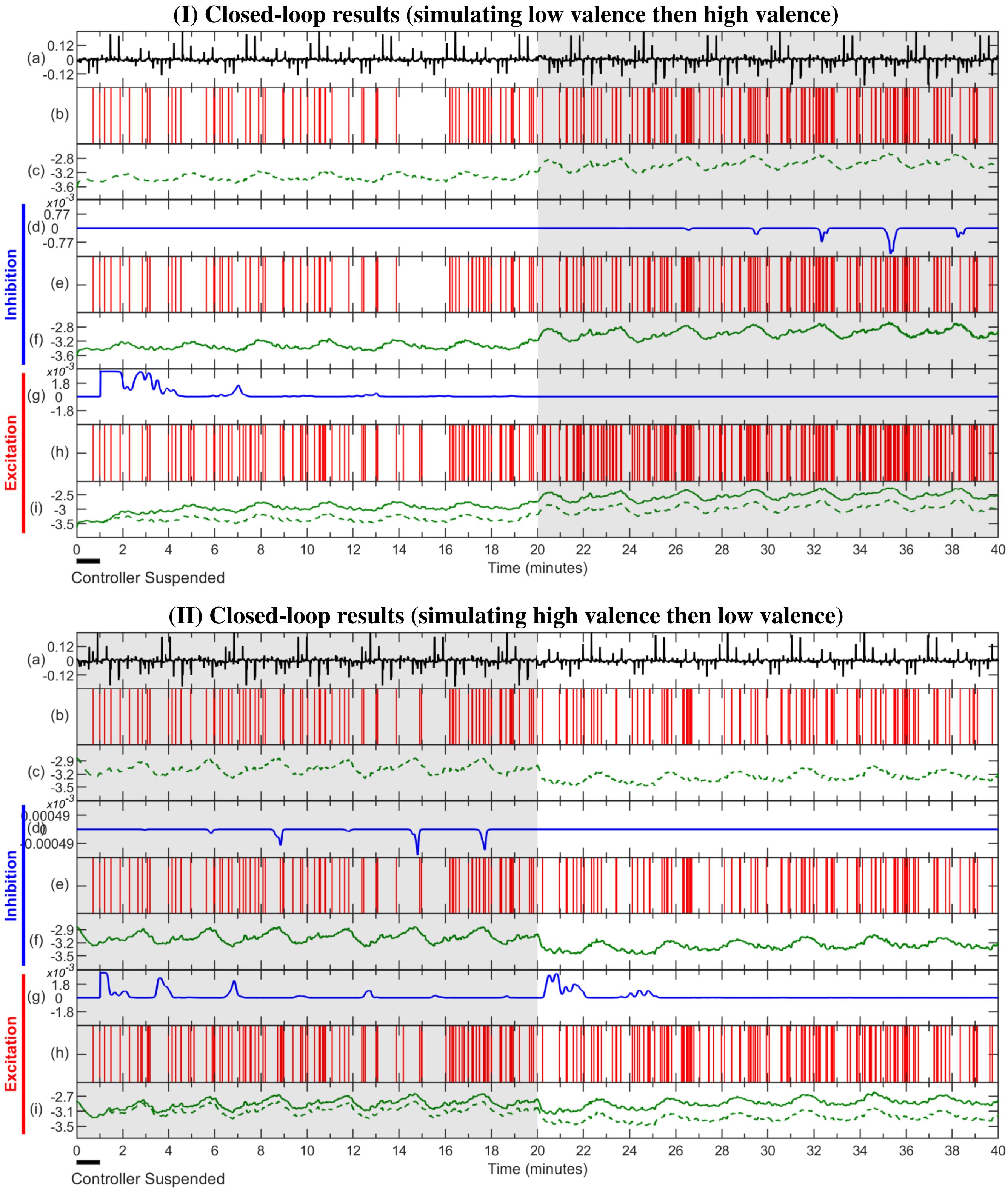

Figure S20. Simulation results of open-loop, inhibitory closed-loop and excitatory closed-loop scenarios for discarded subject 11. In sub-figure I the external stimulus is comprised of half LV, then half HV, with sub-figure II being the opposite. In both I and II, LV and HV periods are represented with unshaded and grey-shaded areas, respectively. Sub-panel (a) depicts environmental stimulus (black) used in all three simulation scenarios. The sub-panels (b) and (c) show spike activity (red) and estimated valence state (green, dashed) during the open-loop, respectively. Sub-panels (d, e, f) display inhibitory closed-loop results, with (d) showing control effort (blue), (e) the corresponding binary signal (red) and (f) the comparison between open-loop (green, dashed) and closed-loop (green, solid) valence state. In a similar fashion, sub-panels (g, h, i) exhibit the excitatory closed-loop outcome.

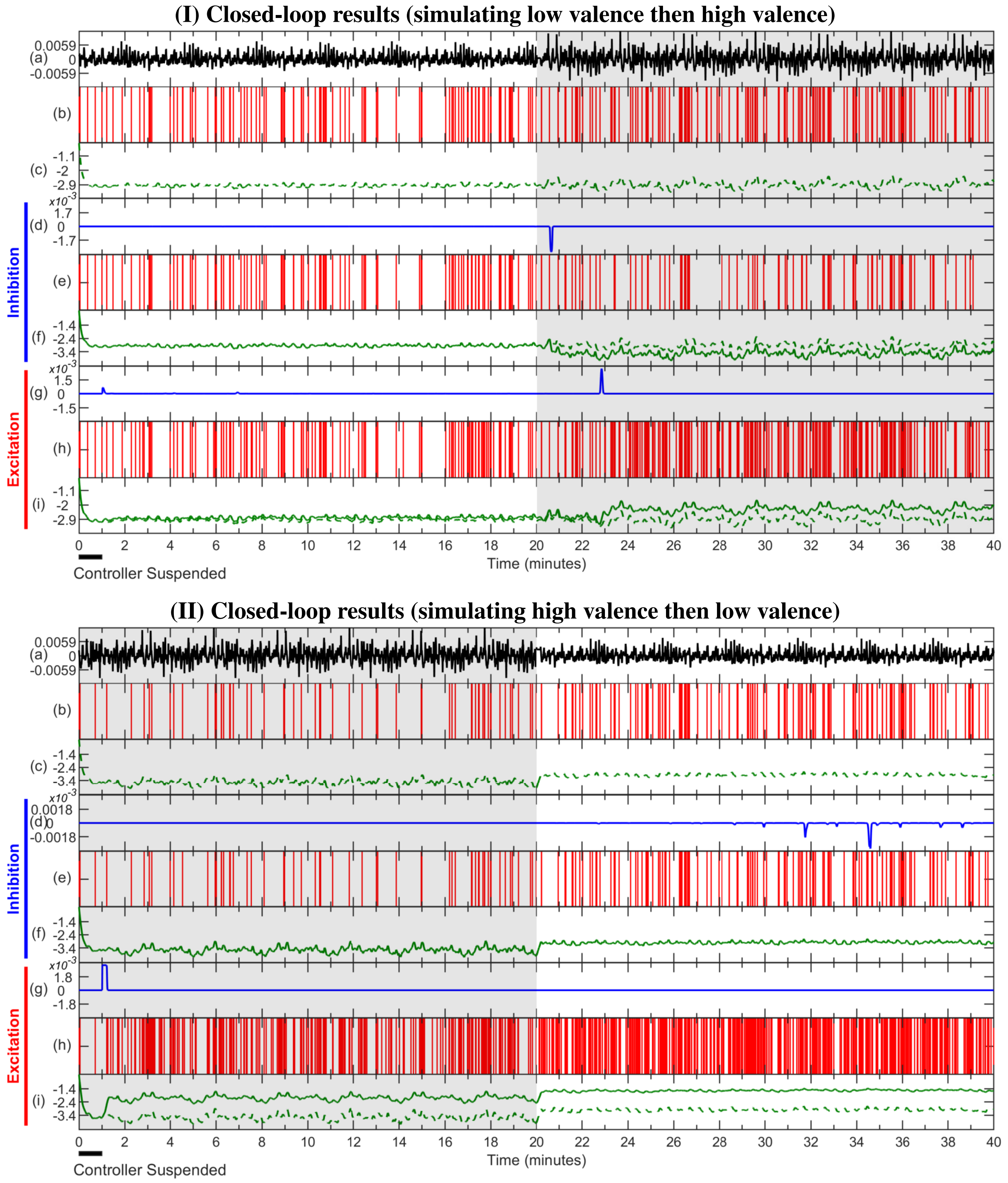

Figure S21. Simulation results of open-loop, inhibitory closed-loop and excitatory closed-loop scenarios for discarded subject 12. In sub-figure I the external stimulus is comprised of half LV, then half HV, with sub-figure II being the opposite. In both I and II, LV and HV periods are represented with unshaded and grey-shaded areas, respectively. Sub-panel (a) depicts environmental stimulus (black) used in all three simulation scenarios. The sub-panels (b) and (c) show spike activity (red) and estimated valence state (green, dashed) during the open-loop, respectively. Sub-panels (d, e, f) display inhibitory closed-loop results, with (d) showing control effort (blue), (e) the corresponding binary signal (red) and (f) the comparison between open-loop (green, dashed) and closed-loop (green, solid) valence state. In a similar fashion, sub-panels (g, h, i) exhibit the excitatory closed-loop outcome.

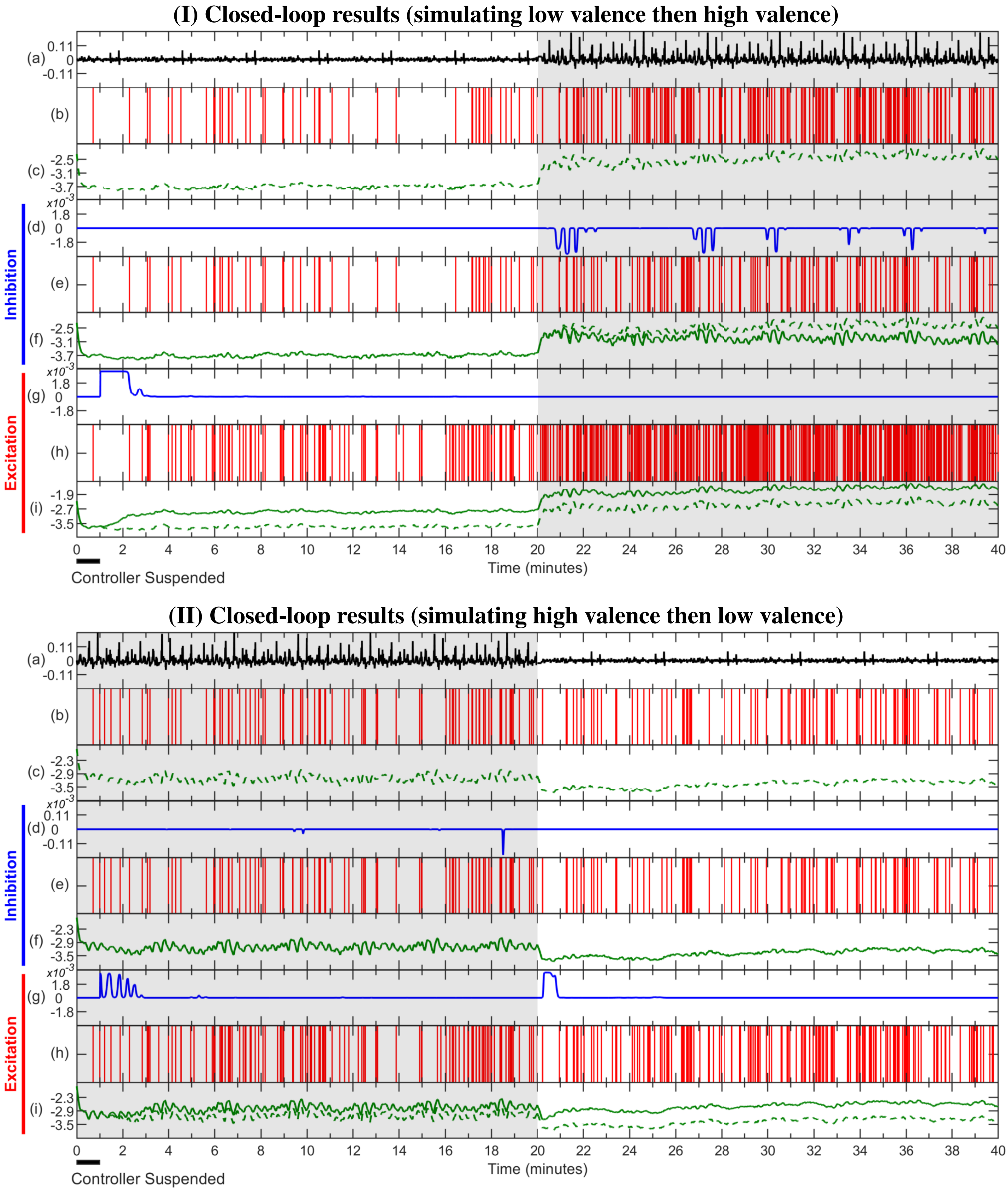

Figure S22. Simulation results of open-loop, inhibitory closed-loop and excitatory closed-loop scenarios for discarded subject 20. In sub-figure I the external stimulus is comprised of half LV, then half HV, with sub-figure II being the opposite. In both I and II, LV and HV periods are represented with unshaded and grey-shaded areas, respectively. Sub-panel (a) depicts environmental stimulus (black) used in all three simulation scenarios. The sub-panels (b) and (c) show spike activity (red) and estimated valence state (green, dashed) during the open-loop, respectively. Sub-panels (d, e, f) display inhibitory closed-loop results, with (d) showing control effort (blue), (e) the corresponding binary signal (red) and (f) the comparison between open-loop (green, dashed) and closed-loop (green, solid) valence state. In a similar fashion, sub-panels (g, h, i) exhibit the excitatory closed-loop outcome.

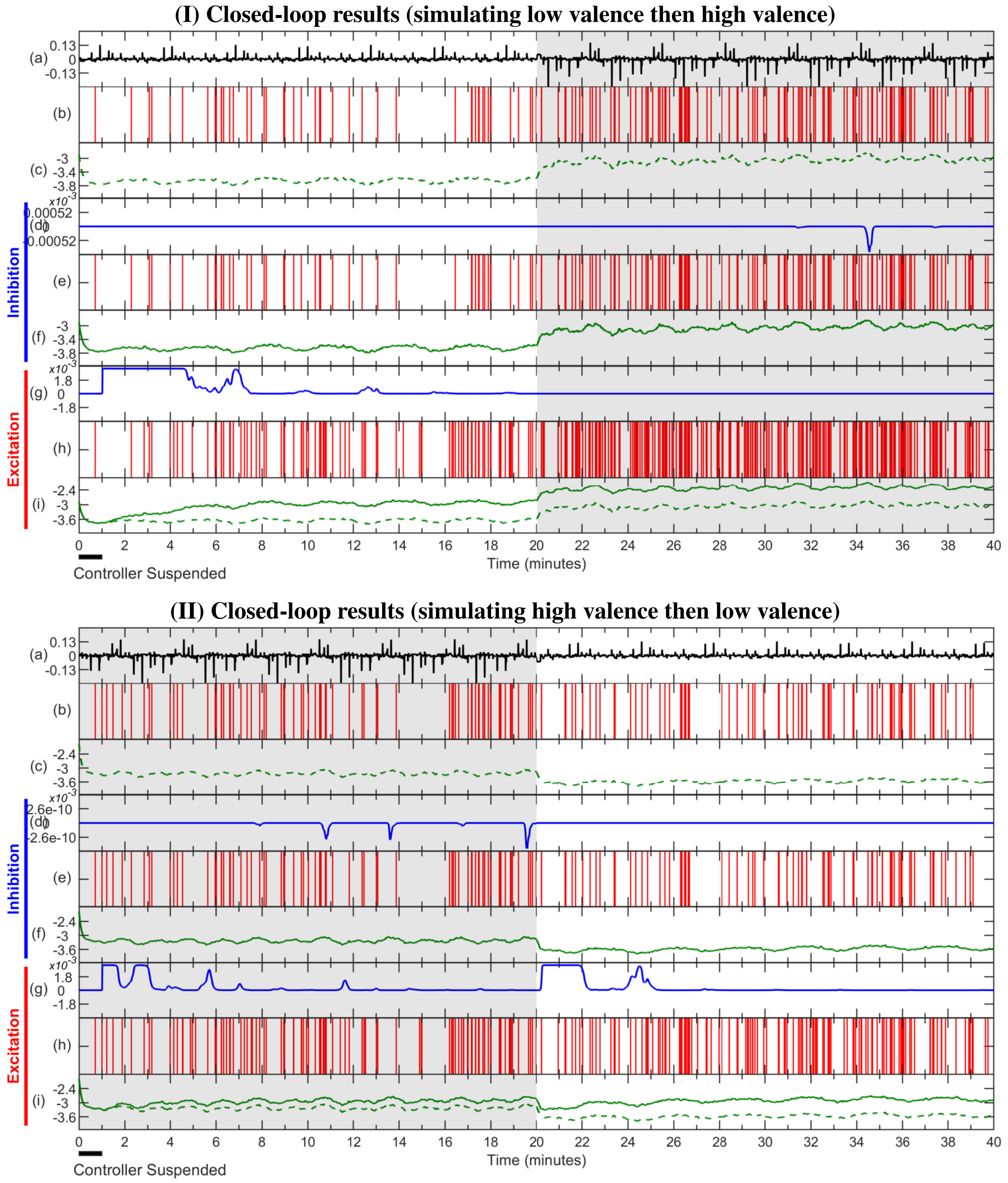

Figure S23. Simulation results of open-loop, inhibitory closed-loop and excitatory closed-loop scenarios for discarded subject 21. In sub-figure I the external stimulus is comprised of half LV, then half HV, with sub-figure II being the opposite. In both I and II, LV and HV periods are represented with unshaded and grey-shaded areas, respectively. Sub-panel (a) depicts environmental stimulus (black) used in all three simulation scenarios. The sub-panels (b) and (c) show spike activity (red) and estimated valence state (green, dashed) during the open-loop, respectively. Sub-panels (d, e, f) display inhibitory closed-loop results, with (d) showing control effort (blue), (e) the corresponding binary signal (red) and (f) the comparison between open-loop (green, dashed) and closed-loop (green, solid) valence state. In a similar fashion, sub-panels (g, h, i) exhibit the excitatory closed-loop outcome.

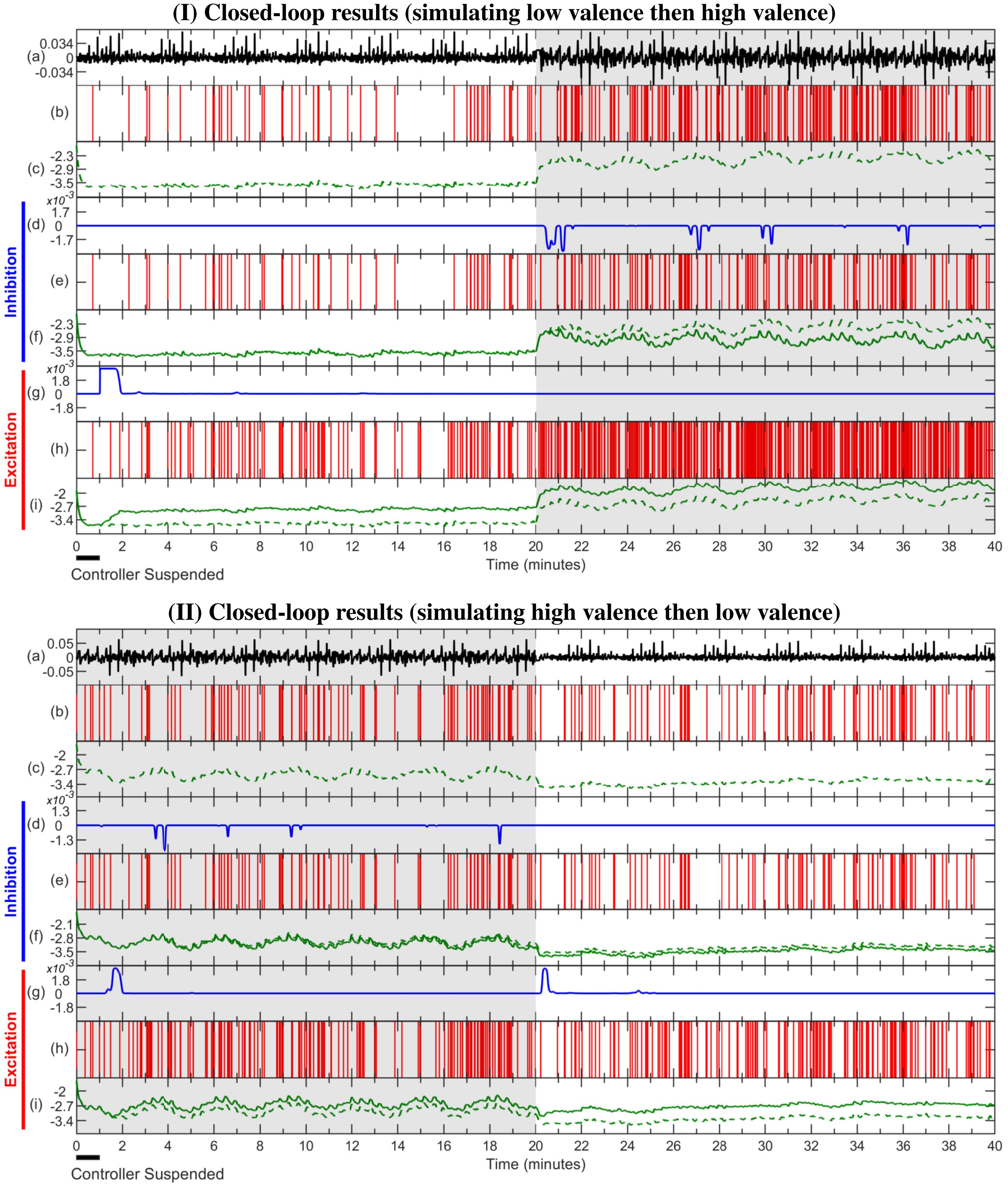

Figure S24. Simulation results of open-loop, inhibitory closed-loop and excitatory closed-loop scenarios for discarded subject 23. In sub-figure I the external stimulus is comprised of half LV, then half HV, with sub-figure II being the opposite. In both I and II, LV and HV periods are represented with unshaded and grey-shaded areas, respectively. Sub-panel (a) depicts environmental stimulus (black) used in all three simulation scenarios. The sub-panels (b) and (c) show spike activity (red) and estimated valence state (green, dashed) during the open-loop, respectively. Sub-panels (d, e, f) display inhibitory closed-loop results, with (d) showing control effort (blue), (e) the corresponding binary signal (red) and (f) the comparison between open-loop (green, dashed) and closed-loop (green, solid) valence state. In a similar fashion, sub-panels (g, h, i) exhibit the excitatory closed-loop outcome.
